# Water extract of cacumen platycladi promotes hair growth through the Akt/GSK3β/β-catenin signaling pathway

**DOI:** 10.3389/fphar.2023.1038039

**Published:** 2023-02-20

**Authors:** Hangjie Fu, Wenxia Li, Zhiwei Weng, Zhiguang Huang, Jinyuan Liu, Qingqing Mao, Bin Ding

**Affiliations:** ^1^ College of Life Science, Zhejiang Chinese Medical University, Hangzhou, China; ^2^ Academy of Chinese Medical Science, Zhejiang Chinese Medical University, Hangzhou, China; ^3^ The Fourth School of Clinical Medicine, Zhejiang Chinese Medical University, Hangzhou, China

**Keywords:** alopecia, cacumen platycladi, hair follicles, dermal papilla cells, Akt, GSK3β

## Abstract

*Cacumen Platycladi* (CP) consists of the dried needles of *Platycladus orientalis* L.) Franco. It was clinically demonstrated that it effectively regenerates hair, but the underlying mechanism remains unknown. Thus, we employed shaved mice to verify the hair growth-promoting capability of the water extract of *Cacumen Platycladi* (WECP). The morphological and histological analyses revealed that WECP application could significantly promote hair growth and hair follicles (HFs) construction, in comparison to that of control group. Additionally, the skin thickness and hair bulb diameter were significantly increased by the application of WECP in a dose-dependent manner. Besides, the high dose of WECP also showed an effect similar to that of finasteride. In an *in vitro* assay, WECP stimulated dermal papilla cells (DPCs) proliferation and migration. Moreover, the upregulation of cyclins (cyclin D1, cyclin-dependent kinase 2 (CDK2), and cyclin-dependent kinase 4 (CDK4)) and downregulation of P21 in WECP-treated cell assays have been evaluated. We identified the ingredients of WECP using ultra-high-performance liquid chromatography-quadrupole time-of-flight mass spectrometry (UPLC-Q/TOF-MS) and endeavored to predict their relevant molecular mechanisms by network analysis. We found that the Akt (serine/threonine protein kinase) signaling pathway might be a crucial target of WECP. It has been demonstrated that WECP treatment activated the phosphorylation of Akt and glycogen synthase kinase-3-beta (GSK3β), promoted β-Catenin and Wnt10b accumulation, and upregulated the expression of lymphoid enhancer-binding factor 1 (LEF1), vascular endothelial growth factor (VEGF), and insulin-like growth factor 1 (IGF1). We also found that WECP significantly altered the expression levels of apoptosis-related genes in mouse dorsal skin. The enhancement capability of WECP on DPCs proliferation and migration could be abrogated by the Akt-specific inhibitor MK-2206 2HCl. These results suggested that WECP might promote hair growth by modulating DPCs proliferation and migration through the regulation of the Akt/GSK3β/β-Catenin signaling pathway.

## 1 Introduction

Hair follicles (HFs) consist of multiple epithelial and dermal papilla cells (DPCs). The latter, which is located at the base of HFs, promotes hair growth and regulates the hair cycle. DPCs content is an indicator of hair growth and dramatically increases during HFs reconstruction (Zhang et al., 2019; Ki et al., 2020). DPCs secrete signaling proteins, such as the wingless-INT (Wnt) family, that promote the proliferation and differentiation of surrounding stromal cells and induce HFs to enter new growth phases (Kim et al., 2016).

The Wnt signaling pathway may regulate multiple processes such as wound skin remodeling, HFs morphogenesis, hair shaft growth, and hair cycle (Kishimoto et al., 2000; Huelsken et al., 2001; Tsai et al., 2014; Sunkara et al., 2022). Wu et al. (2020) demonstrated that Wnt10b overexpression could promote DPC proliferation. Wnt10b and Wnt10a are upregulated in placodes during follicle morphogenesis and postnatal HFs regeneration (Reddy et al., 2001). Huelsken et al. (2001) found that β-Catenin knockdown prevents the differentiation of stem cells into follicular keratinocytes and impairs HFs regeneration. β-Catenin is a major factor in the Wnt signaling pathway (Tsai et al., 2014). It may also regulate epithelial cell differentiation into HFs that generate the hair shaft and inner root sheath. β-Catenin might also enhance the transition from telogen to anagen (Wu et al., 2020).

The Wnt/β-Catenin pathway is a complex signal transduction process wherein phosphorylated GSK3β captures β-Catenin and triggers its degradation. In the presence of Wnt, GSK3β was inactivated and β-Catenin accumulated in the cytoplasm (Liu et al., 2022). However, Akt, also known as protein kinase B, catalyzes GSK3β inactivation as well. The Akt/GSK3β signaling pathway mediates the formation of stabilized dephosphorylated β-Catenin, which is then translocated to the nucleus, where it regulates the transcriptional expression of its target genes (Ki et al., 2020).

In recent years, the global incidence of hair loss and, therefore, the administration of botanical drugs that stimulate DPCs reproduction and activity has gradually increased. CP consists of the dried needles of the evergreen conifer *Platycladus orientalis* (L.) Franco. This tree species is widely distributed worldwide. CP extract possesses antibacterial, anti-inflammatory, and hemostatic activity (Chen et al., 2015; Huang et al., 2020; Zhang et al., 2022). The efficacy of CP against alopecia has been recorded in various ancient Chinese medicine books, such as the Compendium of Materia Medica (Ben Cao Gang Mu in Chinese) and Rihuazi Materia Medica (Ri Hua Zi Ben Cao in Chinese). Current pharmacological studies have shown that CP promotes hair growth and inhibits 5α-reductase activity *in vitro* (Zhang et al., 2013; Zhang et al., 2016; Zhang et al., 2019). However, there have been few published studies on the identity and modes of action of the individual ingredients of CP. In the present study, we evaluated the ability of WECP on hair growth and identified the principal ingredients. We then predicted the regulatory mechanisms of these ingredients by network analysis methods and also elucidated the underlying mechanisms through *in vitro* and *in vivo* experiments.

## 2 Materials and methods

### 2.1 Reagents and biochemicals

MK-2206 2HCl and finasteride (Fin) were purchased from Shanghai Selleck Chemicals Co., Ltd. Shanghai, China. Organic reagents such as ethanol and acetonitrile were obtained from Sinopharm Chemical Reagent Co. Ltd. Shanghai, China. Cell Cycle Assay Kit was acquired from Dojindo, Kumamoto, Japan. Primary antibodies included anti-β-Catenin, anti-GSK3β, anti-phospho-GSK3β, anti-GAPDH, and anti-β-tubulin (Boster Biological Technology, Pleasanton, CA, United States), anti-Bcl2, anti-Bax, and anti-Cyclin D1 (Abcam plc, Cambridge, United Kingdom), anti-Akt and anti-phospho-Akt (Cell Signaling Technology, Danvers, MA, United Ststes), anti-Wnt10b, anti-CDK2, anti-CDK4, and anti-P21 (ABclonal, Wuhan, China), and anti-ki67 (Proteintech, Wuhan, China). The chemiluminescence kit was obtained from Vazyme Biotech, Nanjing, China. The terminal deoxynucleotidyl transferase dUTP nick end labeling (TUNEL) detection kit was acquired from Promega Corporation, Madison, WI, United States.

### 2.2 Cell lines and cell growth

Immortalized DPCs were purchased from Applied Biological Materials Inc. Richmond, BC, Canada, and cultured in Complete Medium (Meisen CTCC, Zhejiang, China) in an incubator (3111; Thermo Fisher Scientific, Waltham, MA, United States) at 37°C, 100% RH, and 5% CO_2_.

### 2.3 WECP preparation

CP was procured from the Chinese Herbal Pieces Factory of Zhejiang Chinese Medical University, Hangzhou, China. We used dried botanical drugs (100 g) for circumfluence extraction, which was carried out twice in 1.5 L boiling distilled water for 1 h each time. The supernatant was collected and concentrated in a rotary vacuum evaporator (R-100; BUCHI, Switzerland). The extract was lyophilized (100-9; LaboGene, Lillerød, Denmark), and 15.287 g powder product was stored at −20°C until further analysis.

### 2.4 Analysis and identification of secondary metabolites in WECP by UPLC-Q/TOF-MS

The UPLC-Q/TOF-MS system was used to measure the mass of each secondary metabolites in the WECP. The dried WECP powder was reconstituted in desalinated water to obtain 1 mg/mL WECP aqueous solution. Then 2 μL was injected into a CORTECS UPLC T3 column (2.1 mm × 100 mm; 1.6 μm) (Waters Corp. Milford, MA, United States) fitted with the UPLC system (Waters Corp.). The mobile phase was a mixture of acetonitrile (A) and 0.1% (v/v) formic acid B). The elution program was set as follows: 0–2.00 min, 5% A; 2.01–32.00 min, 5%–100% A; and 32.01–35.00 min, 5% A. The flow rate was set as 0.3 mL/min. The mass of each ingredient was measured by SYNAPT G2-Si ion mobility mass spectrometry (Waters Corp.). Electrospray ionization mass spectrometry was performed in positive and negative modes. The mass-to-charge ratio (m/z) scan range was 50–1,200. Finally, the results were comparatively analyzed using SCIEX OS software.

### 2.5 Network analysis

The putative pharmacological activity of each ingredient identified in WECP was predicted by network analysis.

#### 2.5.1 Prediction of targets of secondary metabolites in WECP

The ingredients in WECP were identified with UPLC-Q/TOF-MS and compaired with those of CP, which were collected from the Traditional Chinese Medicine Systems Pharmacology Database (TCMSP, http://tcmspw.com). The ingredients were finally select with parameters, oral bioavailability (OB) > 30%, drug-likeness (DL) > 0.18. SDF files for the 3D structures of the ingredients in WECP were downloaded from the PubChem database (https://pubchem.ncbi.nlm.nih.gov/). Putative targets of each ingredient were identified on the PharmMapper (http://www.lilab-ecust.cn/pharmmapper/) platform. Duplicate items were deleted.

#### 2.5.2 Determination of potential hair loss targets

The DisGeNET (https://www.disgenet.org/) and GeneCards (https://www.genecards.org/) databases were used to collect genes associated with hair loss. The genes were collected with parameter: score_gad ≥ 0.1 in DisGeNET and relevance score ≥ 10 in GeneCard. Candidate gene targets of the ingredients in WECP were obtained using the Venn 2.1.0 online platform (http://bioinformatics.psb.ugent.be/webtools/Venn/) and visualized as a network with Cytoscape v. 3.7.1 (https://nrnb-nexus.ucsd.edu/repository/cytoscape_releases/).

#### 2.5.3 Construction of the protein-protein interaction (PPI) network

Complex interactions among potential targets were analyzed with the STRING database (https://string-db.org/). The species was set as “*Homo sapiens*,” and the PPI network was visualized with Cytoscape v. 3.7.1.

#### 2.5.4 Gene ontology (GO) and kyoto encyclopedia of genes and genomes (KEGG) pathway enrichment analyses

The putative roles of WECP in the molecular function, cell component, and biological process domains as well as the implicated signaling pathways, were annotated by GO and KEGG pathway analyses in the DAVID database (https://david.ncifcrf.gov/).

### 2.6 Cell proliferation/cytotoxicity assays

Immortalized DPCs were seeded in 96-well plates at a density of 2 × 10^4^/well, grown overnight, and subjected to 10–640 μg/mL WECP for 24 h. DPC viability was then quantified by the CCK-8 method (Jiang et al., 2019). Ten percent CCK-8 (Dojindo, Kumamoto, Japan) was pipetted into the DPC cultures, and the suspensions were incubated in the dark at 37°C for 1 h. The mixtures were then allowed to shake on an automatic rocker (MH-2; Kylin-Bell Lab Instruments Co., Ltd. China) at room temperature for 3 min, and the absorbance of each well was read at 450 nm in a FLUOstar^®^ Omega microplate reader (BMG LABTECH, Offenburg, Germany). In another assay, 5 µM MK-2206 2HCl (Akt inhibitor) was applied to DPCs for 1 h before treatment with WECP. The cells were then incubated for 24 h in fresh Complete Medium containing 160 μg/mL WECP. DPC viability was assessed by CCK-8 assay once again.

### 2.7 Cell cycle analysis by flow cytometry

The DPCs cycle was detected by propidium iodide (PI) staining and flow cytometry (Li et al., 2022). Briefly, DPCs were seeded in six-well plates at a density of 1.5 × 10^5^/well and treated with various WECP concentrations for 24 h. The following day, the cells were harvested, washed with phosphate-buffered saline (PBS), and centrifuged (5425 R; Eppendorf, Germany). The cell pellets were resuspended and fixed with 75% (v/v) pre-chilled ethanol at 4°C for 24 h. The fixed cells were harvested by centrifugation and stained with 50 μg/mL PI in PBS plus 50 μg/mL RNase A at 37°C for 30 min. Twenty thousand cells per well were processed in a BD Accuri C6 flow cytometer (BD Biosciences, San Jose, CA, United States) and analyzed by FlowJo v. 10.0.7r2 software (FlowJo LLC, Ashland, OR, United States).

### 2.8 Wound healing assay

DPCs were seeded in six-well plates at a density of 1.5 × 10^5^/well and grown overnight to confluence. Wounds were gently induced on the monolayer with a 1,000-μL pipette tip. Cell debris was eliminated. Fresh medium containing 1% (v/v) fetal bovine serum plus various WECP concentrations was added to the wells. The plates were stored in a 5% CO_2_ incubator at 37°C overnight. The cells in each plate were pretreated with MK-2206 2HCl for 1 h before the WECP treatments. Identity fields were photographed at each time point, and the healed areas were measured with ImageJ software (National Institutes of Health, Bethesda, MD, United States).

### 2.9 RNA extraction and real-time quantitative polymerase chain reaction (RT-qPCR)

Gene transcription was quantified by RT-qPCR. Briefly, total RNA was prepared with Trizol reagent (Sangon Biotech Co., Ltd. Shanghai, China) according to the manufacturer’s instructions. The concentration and quality of the isolated RNA were determined by NanoDrop spectrophotometry (Thermo Fisher Scientific). RT-qPCR was performed with iQ-SYBR Green PCR Supermix (Bio-Rad Laboratories, Hercules, CA, United States) and specific primes pairs in a StepOnePlus RT-qPCR system (Thermo Fisher Scientific). The primer sequences are listed in Table 1. Relative gene expression was calculated by the 2^−ΔΔCT^ method using GAPDH as the internal reference gene (Schmittgen and Livak, 2008).

**TABLE 1:** The primer pairs.

Gene	Forward (5′−3′)	Reverse (5′−3′)
Wnt5a-M	CAA​CTG​GCA​GGA​CTT​TCT​CAA	CCT​TCT​CCA​ATG​TAC​TGC​ATG​TG
Wnt10b-M	GCG​GGT​CTC​CTG​TTC​TTG​G	CCG​GGA​AGT​TTA​AGG​CCC​AG
β-catenin-M	ATG​GAG​CCG​GAC​AGA​AAA​GC	CTT​GCC​ACT​CAG​GGA​AGG​A
Bcl2-M	GTC​GCT​ACC​GTC​GTG​ACT​TC	CAG​ACA​TGC​ACC​TAC​CCA​GC
Bax-M	TGA​AGA​CAG​GGG​CCT​TTT​TG	AAT​TCG​CCG​GAG​ACA​CTC​G
LEF1-M	TGT​TTA​TCC​CAT​CAC​GGG​TGG	CAT​GGA​AGT​GTC​GCC​TGA​CAG
VEGFA-M	CTG​CCG​TCC​GAT​TGA​GAC​C	CCC​CTC​CTT​GTA​CCA​CTG​TC
IGF1-M	CTG​GAC​CAG​AGA​CCC​TTT​GC	GGA​CGG​GGA​CTT​CTG​AGT​CTT
GAPDH-M	AGG​TCG​GTG​TGA​ACG​GAT​TTG	TGT​AGA​CCA​TGT​AGT​TGA​GGT​CA
LEF1-H	AGA​ACA​CCC​CGA​TGA​CGG​A	GGC​ATC​ATT​ATG​TAC​CCG​GAA​T
VEGF-H	AGG​GCA​GAA​TCA​TCA​CGA​AGT	AGG​GTC​TCG​ATT​GGA​TGG​CA
IGF-1-H	GCT​CTT​CAG​TTC​GTG​TGT​GGA	GCC​TCC​TTA​GAT​CAC​AGC​TCC
GAPDH-H	CTG​GGC​TAC​ACT​GAG​CAC​C	AAG​TGG​TCG​TTG​AGG​GCA​ATG

### 2.10 Western blot

Western blotting was performed according to a previously reported method (Ma et al., 2019). Mouse dorsal skin tissue or cultured DPCs were lysed in radioimmunoprecipitation assay buffer containing protease and phosphatase inhibitors (CWBIO, Jiangsu, China) to release the proteins. The latter were quantified with a bicinchoninic acid assay kit (Beyotime Biotechnology Inc. Shanghai, China) according to the manufacturer’s instructions. Equal amounts of protein lysates were separated by 10% or 12% sodium dodecyl sulfate-polyacrylamide gel electrophoresis gel electrophoresis. For specific antibody hybridization detection, the separated proteins were transferred to polyvinylidene difluoride membranes (EMD Millipore Corporation, Billerica, MA, Unites States) which were then blocked with 5% (v/v) non-fat milk for 1 h and incubated with primary antibodies at 4°C overnight. The following day, the membranes were washed thrice with 0.1% (v/v) Tris-buffered saline with Tween-20. The membranes were then separately incubated with the corresponding secondary antibodies, subjected to an enhanced chemiluminescence kit, and observed under ChemiScope 6000 Series Chemiluminescence Imaging System (Clinx Science Instrument Co. Ltd. Shanghai, China) according to the manufacturer’s instructions.

### 2.11 Hair loss mouse model establishment and trial grouping

The anesthetized C57BL/6 mice (male, age 7 weeks, 20 ± 2 g) were then shaved with a clipper (Codos; Shenzhen, China), denuded with depilatory cream (Reckitt Benckiser, Hubei, China), and randomly divided into four groups (n = 6), namely, saline control (intragastrically administered 200 μL saline); low-dose WECP group (WECPL; intragastrically administered 160 mg/kg WECP in 200 μL saline); high dose WECP (WECPH; intragastrically administered 320 mg/kg WECP in 200 μL saline); and Fin (intragastrically administered 10 mg/kg Fin in 200 μL saline). All mice were administered their respective treatments daily for 20 days. They were also anesthetized and photographed on days 0, 10, and 20. After the experiment, all mice were sacrificed to obtain skin tissue samples for subsequent analysis. All animal experimentation was approved by the Animal Experimental Ethics Committee of Zhejiang Chinese Medical University under approval No. IACUC-20220214–21.

### 2.12 Morphological and histological study

Mouse dorsal skin tissues were fixed in 4% (v/v) formaldehyde, embedded in paraffin, sliced into 5-µm sections (HistoCoreBIOCUT; Leica Camera AG, Germany), and subjected to hematoxylin and eosin (H&E) staining (Liu et al., 2022). Skin thickness and dermal papilla diameters were measured with Slideviewer software (NDP view2; Beijing, China). A TUNEL assay and ki67 immunofluorescence detection were used to evaluate HFs apoptosis and proliferation. For the TUNEL assay, the slides were dewaxed, rehydrated, deproteinized, washed, incubated in TUNEL solution at 37°C for 1 h, and visualized with horseradish peroxidase-conjugated secondary antibody solution. The cell nuclei were stained with hematoxylin. All sections were observed and imaged with a digital pathology scanner (VS120-S6-W; Olympus, Tokyo, Japan). For the ki67 immunofluorescence detection, the rehydrated slides were heated and subjected to the ki67 antigen retrieval solution in the kit. The slides were blocked using a blocking buffer to avoid non-specific hybridization. The ki67 was then hybridized with a specific antibody and detected using a fluorescent reagent. The nuclei were stained with 4′,6-diamidino-2-phenylindole and imaged by fluorescence microscopy (ECLIPSE C1; Nikon Corporation, Tokyo, Japan).

### 2.13 Statistical analyses

All data are represented as means ± standard deviation (SD) of ≥ 3 independent biological experiments and three replicates of each experiment. Statistical analyses were performed in SPSS v. 16.0 (IBM Corp. Armonk, NY, United States). Pairwise differences between treatments were analyzed by one-way ANOVA, and a *post hoc* Fisher’s least significant difference test was used. Differences between treatments were considered statistically significant if *p* < 0.05.

## 3 Result

### 3.1 WECP promoted hair growth and HFs reconstruction in mice

**FIGURE 1:**
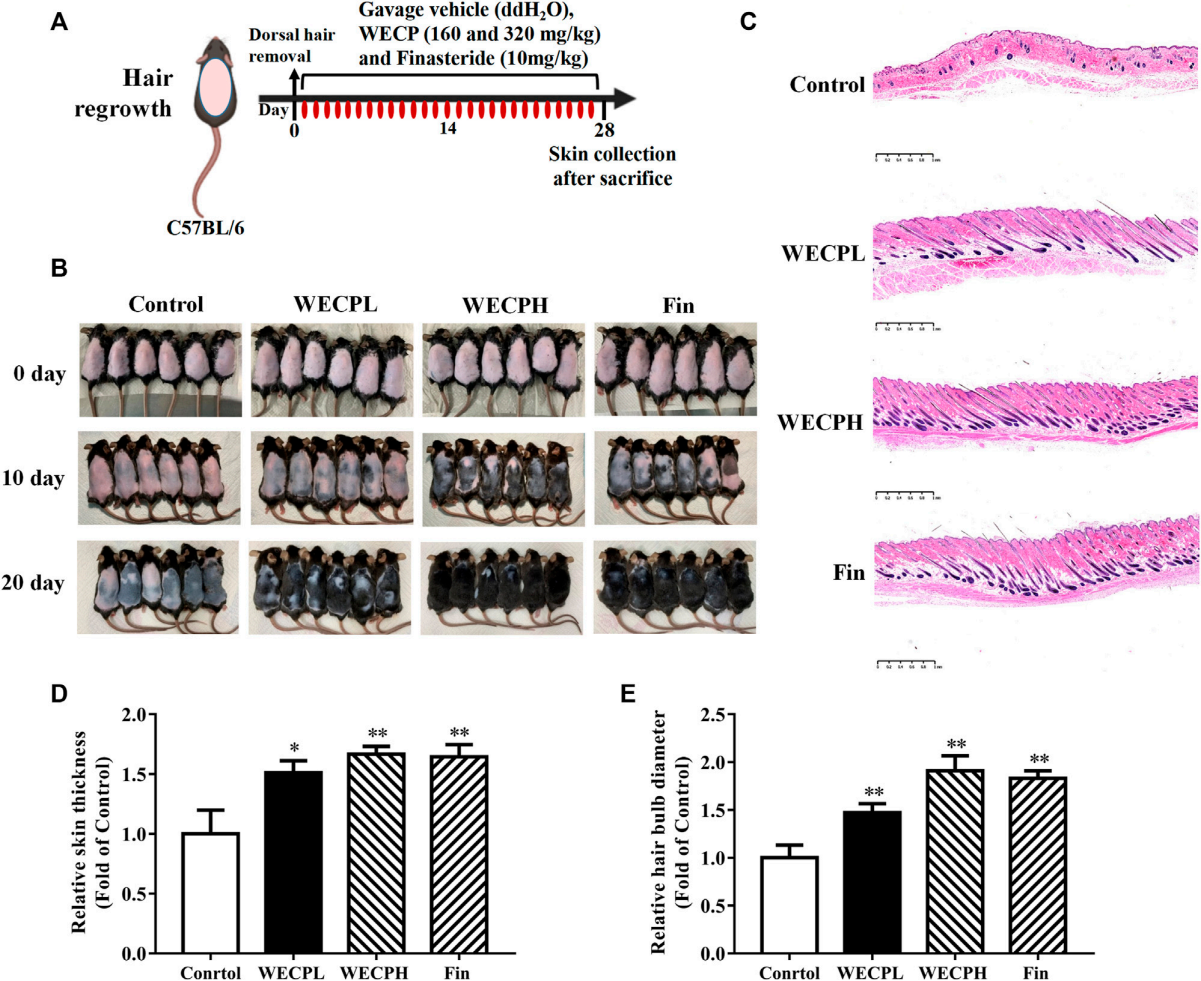
WECP promotes hair regrowth *in vivo*. **(A)** Back skin of 7-week-old C57BL/6 mice was shaved. WECP (160 mg/kg and 320 mg/kg) and finasteride (10 mg/kg) were administered daily by gavage for 20 days. **(B)** Photographs of dorsal skin on days 10 and 20 after depilation. **(C)** Depilated mouse skin tissue sections were prepared and histologically examined using H & E staining. Images were captured using a digital pathology scanner. **(D, E)** Skin thickness and hair bulb diameter were measured. N = 6. Scale bars = 1 mm. WECPL, WECPH, and Fin represent low-dose WECP, high-dose WECP, and finasteride treatment, respectively. *p < 0.05, **p < 0.01 vs. control group.

We used 7 weeks old male C57BL/6 mice to evaluate the ability of WECP to stimulate hair growth. The HFs were in the telogen phase (Figure 1A). We observed rapid hair growth in the WECP and Fin groups compared with that in the denuded control (Figure 1B). The HFs and dermis were observed under a microscope after H&E staining to explore the effect of WECP. Compared with the control, there were significantly more HFs, and the hair shafts had grown through the dermis and epidermis in the WECP and Fin groups (Figure 1C). The hair bulb size and dermis and epidermis thickness were significantly increased in the WECP and Fin groups than those in the control (Figures 1D, E). Moreover, the quantitative analysis indicated that WECP stimulated hair growth in a dose-dependent manner. To our surprise, the high dose WECP showed similar efficacy to Fin.

### 3.2 WECP stimulated DPCs proliferation

**FIGURE 2:**
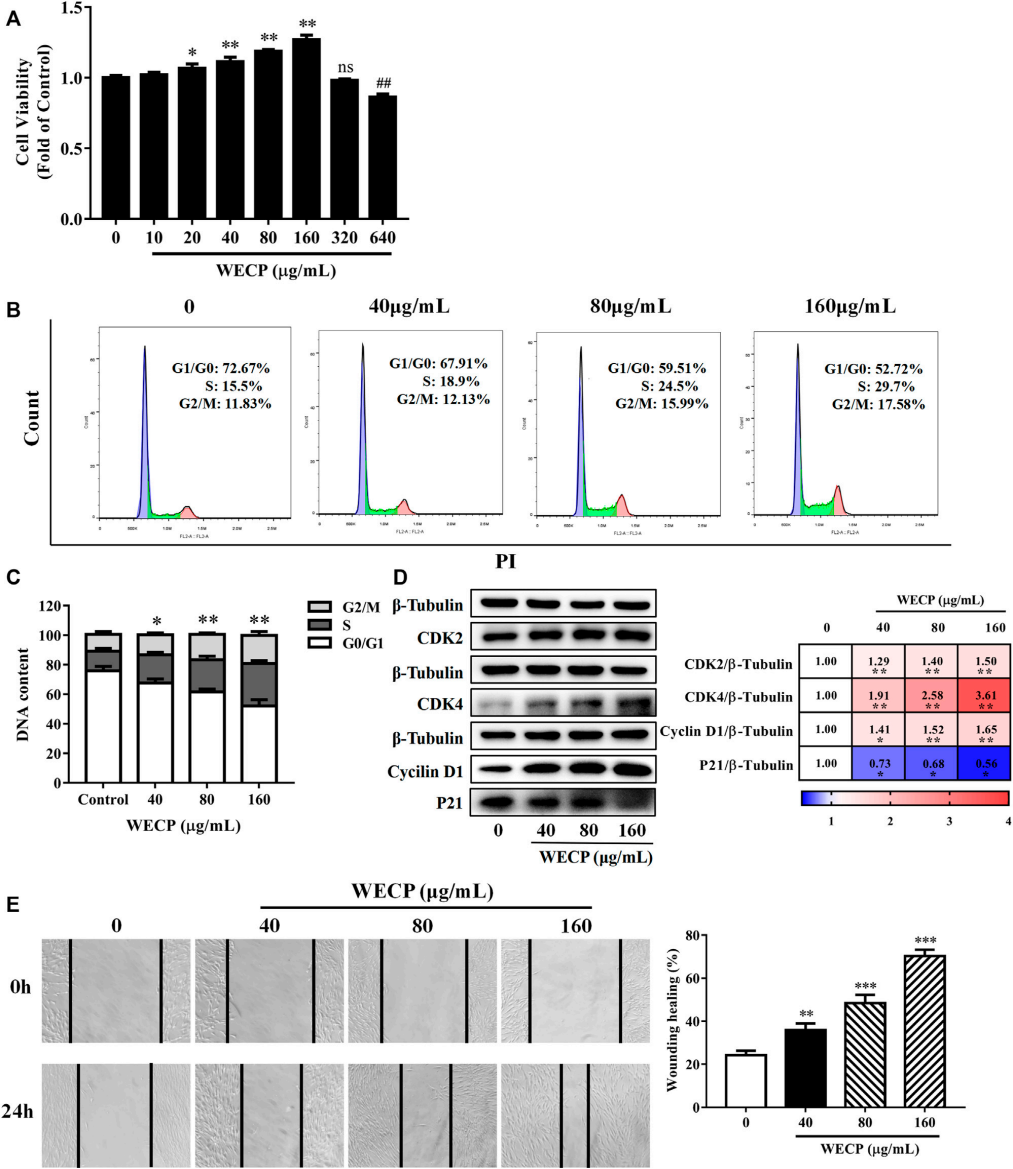
WECP increased DPC proliferation and migration. **(A)** Cell proliferation was measured using the CCK-8 assay. DPCs were treated with various concentrations of WECP (10, 20, 40, 80, 160, 320 and 640 μg/mL). Data were means of three independent experiments. Error bars represent standard deviation (SD). *p < 0.05, and **p < 0.01 vs. vehicle-treated control. DPCs were treated with WECP (40, 80, and 160 μg/mL) for 24 h. **(B)** Distribution of DPCs in G1, S, and G2 after WECP treatment detected using flow cytometry and **(C)** quantitatively analyzed using FlowJo v. 10.0.7r2. **(D)** Expression of cell cycle-related proteins was determined by western blotting after WECP treatment. **(E)** Effects of 24 h WECP treatment on DPC migration were observed and quantified. Data were means ± SD of three independent experiments. *p < 0.05, **p or ##*p* < 0.01 and ***p < 0.001 vs. control. NS, not significant.

DPCs occur in HFs and play important roles in the hair growth cycle. Previous studies suggested that DPC proliferation influences HFs reconstruction and the hair growth cycle (Yoon et al., 2014; Cao et al., 2021). We evaluated the effects of various WECP concentrations on the DPCs viability and cell cycle. DPCs proliferated significantly faster after WECP treatment and especially at 160 μg/mL. However, WECP was cytotoxic to DPCs at 640 μg/mL (Figure 2A).

### 3.3 WECP accelerated the DPCs cell cycle

Mice were sacrificed after the experiment and their back skin was subjected to histopathological analysis to explore the effects of WECP on HFs and the dermis. The number of HFs had significantly increased, and the HF structure was significantly completed after WECP and Fin treatment (Figure 1C). The cell cycle distribution of the DPCs was assessed by flow cytometry to determine whether WECP causes DPCs proliferation through cell cycle progression. There were significantly fewer cells in G0/G1 phase after 24 h of WECP treatment than there were in the control. Moreover, the number of cells in S phase had significantly increased after WECP treatment in a dose-dependent manner. Hence, the WECP treatment promoted cell cycle progression (Figures 2B, C). The proteins, Cyclin A, CDK2, CDK4, and P21, regulate cell cycle progression from G0/G1 to S (Li et al., 2022). We then quantified these cell cycle-related proteins by western blotting to verify the foregoing results. WECP significantly upregulated cyclin D1, CKD2, and CDK4 proteins and significantly downregulated P21 protein. Thus, WECP promoted cell cycle progression (Figure 2D). The preceding results suggested that WECP accelerates cell cycle progression, thereby promoting DPCs proliferation and hair growth.

### 3.4 WECP promoted DPCs migration

Cell migration is critical in HFs development (Ridley et al., 2003). WECP significantly improved wound healing in a dose-dependent manner (Figure 2E).

### 3.5 Compositional analysis of WECP

We obtained 15.287 g WECP powder by the circumfluence extraction method (Hong et al., 2022). The chemical compounds of WECP were identified by UPLC-Q/TOF-MS in positive and negative ion modes. We obtained 14 and 6 components at the two modes, respectively. Rutin, myricetin, quercetin, and isoquercitrin were observed in both models. The 16 compounds are listed in Supplementary Figure S1; Supplementary Table S1.

### 3.6 Prediction of the possible mechanisms of WECP in alopecia treatment *via* network analysis

**FIGURE 3:**
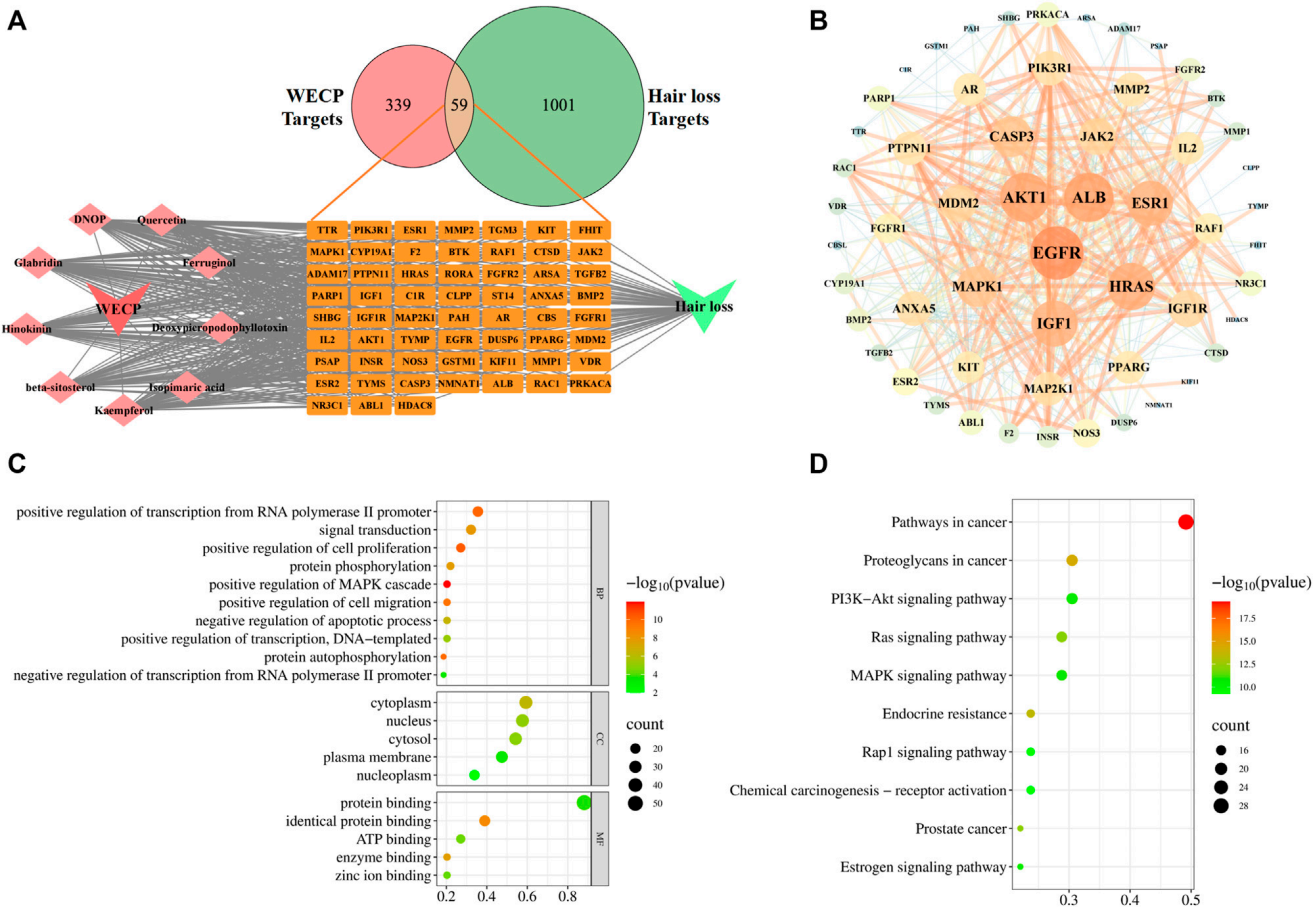
Network analysis of WECP. **(A)** Venn diagram showing 59 predicted WECP targets in hair loss and ingredients-targets-disease network of WECP. **(B)** PPI network of 59 predicted targets visualized with Cytoscape v. 3.7.1. Target size and color are based on degree values. Functional enrichment analysis of WECP. **(C)** GO enrichment analysis of 59 predicted targets. **(D)** KEGG enrichment analyses of 59 predicted targets. Horizontal axis represents the proportion of enriched genes in terms. Vertical axis represents each term. Dot size and color represent the number of enrichment targets and *p*-values, respectively.

We collected nine ingredients in WECP with OB > 30% and DL > 0.18 (Supplementary Table S2). A total of 398 targets for the corresponding nine ingredients in WECP and 1,060 genes (Supplementary Table S3) involved in the hair loss process *via* database screening (Figure 3A). Of these, 59 genes were candidate targets of WECP involved in hair loss prevention. They were then uploaded to the STRING database (https://cn.string-db.org), and those with a confidence value > 0.4 were selected. Complex interactions among these proteins and their associated regulators were illustrated in a PPI network containing 56 nodes and 426 edges (Figure 3B). EGFR, Akt1, ALB, HRAS and IGF1 had the highest node degree values and edge densities.

GO and KEGG analyses of the 59 aforementioned candidate targets indicated that the biological process (BP) terms of the target genes were mainly involved in the positive regulation of cell proliferation (GO:0008284), protein phosphorylation (GO:0006468), positive regulation of cell migration (GO:0030335), and negative regulation of apoptosis (GO:0043066). The cellular component (CC) terms of the target genes were enriched in the cytoplasm (GO:0005737), nucleus (GO:0005634), and cytosol (GO:0005829). The molecular function (MF) terms of the target genes were associated with protein binding (GO:0005515), identical protein binding (GO:0042802), and ATP binding (GO:0005524). The top 10 BP terms and the top 5 CC and MF terms are illustrated in bubble charts, in which the pathway with the more involved genes was shown in the larger bubble (Figure 3C). The top 20 pathways ranked by the gene enrichment are also visualized (Figure 3D). The cancer-related and PI3K/Akt signaling pathways were the most highly enriched.

The PI3K/Akt signaling pathway has been thoroughly studied and plays a vital role in cell proliferation and survival. Phosphorylated Akt can, in turn, phosphorylate its downstream target proteins, such as the GSK-3β and FoxO3a transcription factors (Jiang et al., 2019; Lin et al., 2021). Bae et al. (2022) reported that activated Akt promotes the growth of hair follicle mesenchymal stem cells (HFMSCs) by inhibiting GSK3β, stabilizing β-Catenin, promoting the translocation of the latter into the nucleus, and inducing DPC proliferation. Based on the results of network analysis and the literature review, we hypothesized that WECP promotes hair growth by activating the Akt/GSK3β/β-Catenin signaling axis.

### 3.7 WECP promoted β-Catenin accumulation by activating Akt/GSK3β phosphorylation

**FIGURE 4:**
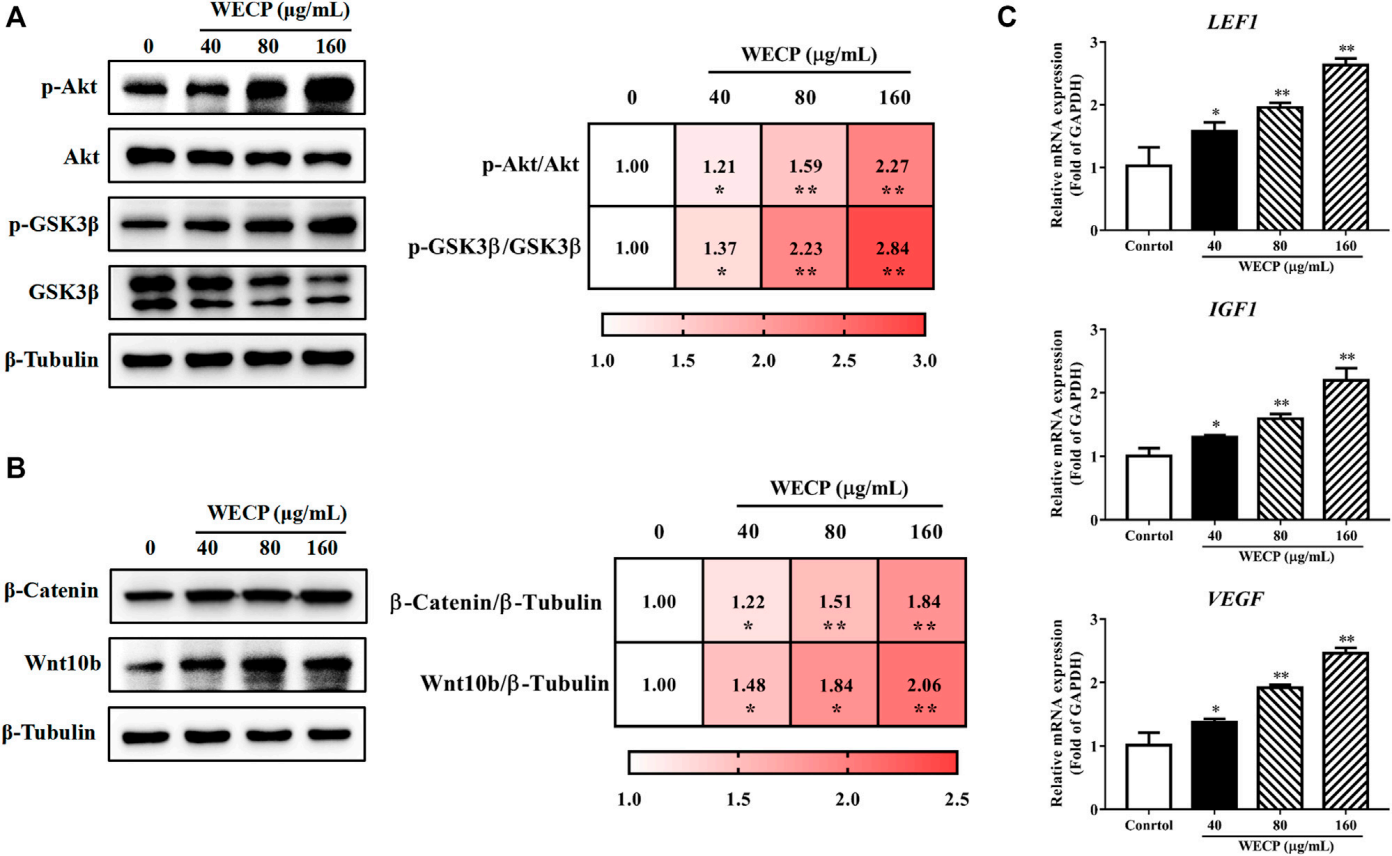
WECP activated Akt/GSK3β/β-Catenin signaling pathway in DPCs. DPCs were incubated with WECP (40, 80 and 160 μg/mL) in culture medium for 24 h. **(A)** Akt and GSK3β phosphorylation levels were detected in DPCs after WECP treatment. **(B)** Effects of WECP treatment on β-Catenin and Wnt10b protein expression levels in DPCs. **(C)** Transcriptional expression of LEF1, IGF1, and VEGF in DPCs detected using RT-PCR. Data are presented as means ± SD of three independent replicates. *p < 0.05, **p < 0.01 vs. control group.

To verify whether WECP promoted hair growth and DPCs proliferation through the Akt/GSK3β/β-Catenin signaling axis, we detected the phosphorylation levels of Akt and GSK3β by western blotting. We found that the phosphorylation level of Akt and GSK3β was upregulated in a dose-dependent manner after WECP treatments (Figure 4A). Then, as we anticipated, activation of Akt inhibited GSK3β activity, promoted β-Catenin accumulation, and elevated protein expression levels of Wnt10b (Figure 4B). Myung et al. (2013) showed that the activation of β-Catenin could enhance the LEF1 transcription factor and contribute to the secretion of VEGF and IGF1, as well as to the growth cytokine enrichment. Therefore, we examined the transcriptional expression of LEF1, VEGF, and IGF1. The results showed that WECP promoted the mRNA expression of these growth factors in DPCs in a dose-dependent manner (Figure 4C).

**FIGURE 5:**
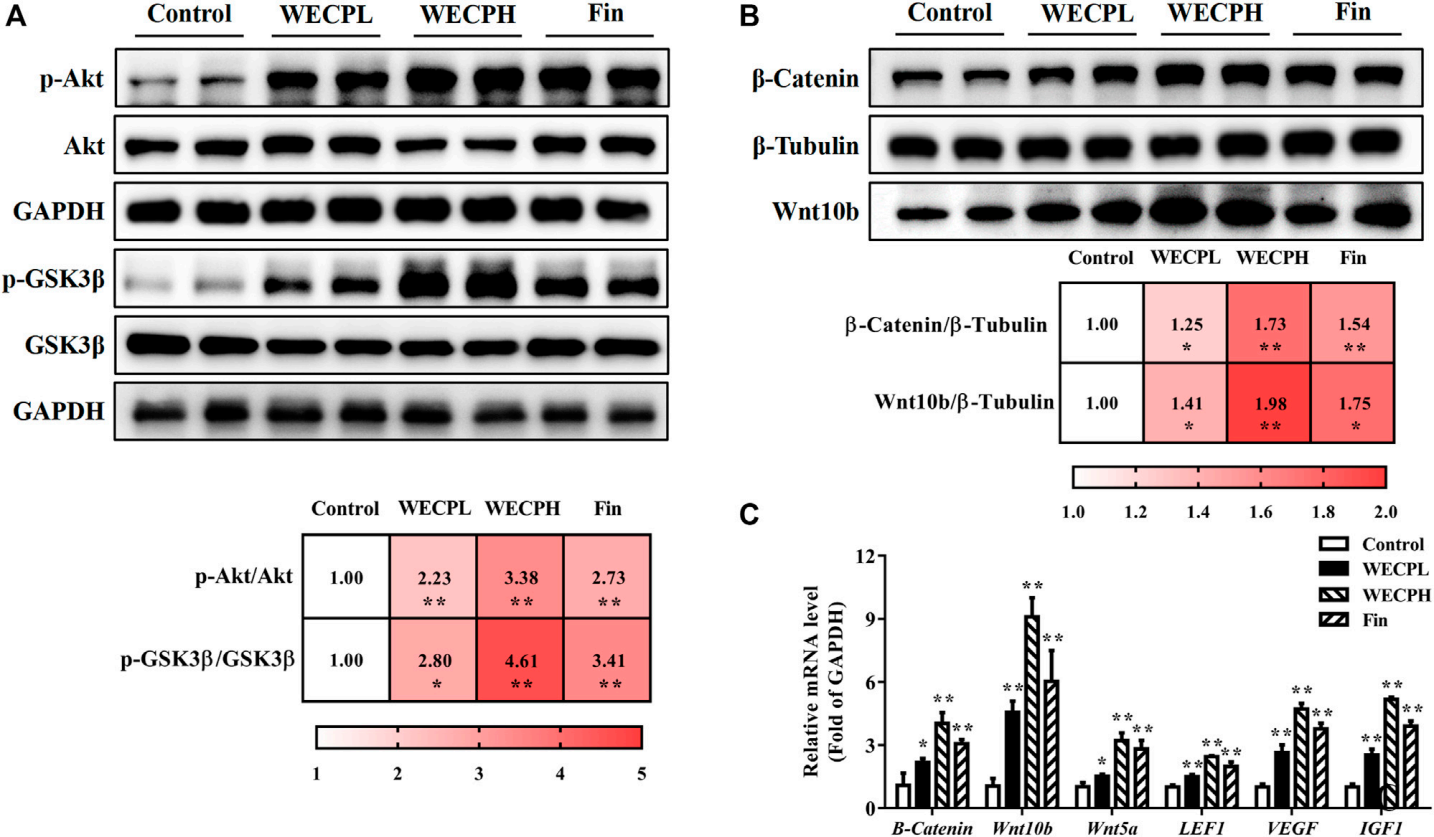
WECP activated Akt/GSK3β/β-Catenin signaling pathway in denuded mouse skin. **(A)** Effects of WECP and finasteride treatments on Akt and GSK3β protein phosphorylation in mouse skin. **(B)** Effects of WECP and finasteride treatments on β-Catenin and Wnt10 translational expression in mouse skin. **(C)** Effects of WECP and finasteride treatments on transcriptional expression of β-Catenin, Wnt10b, Wnt5a, LEF1, VEGF, and IGF1 in mouse skin. Data are means ± SD of three independent replicates. *p < 0.05, **p < 0.01, ***p < 0.001 vs. control group. WECPL, WECPH, and Fin represent low-dose WECP, high-dose WECP, and finasteride treatment, respectively.

To confirm the results at the cellular level, we also detected the phosphorylation levels of Akt and GSK-3β in the back skin of each group (Figure 5A), as well as the translational expression of β-Catenin and Wnt10b (Figure 5B). Consistent with the *in vitro* results, the phosphorylation levels of Akt and GSK3β and the expressions of β-Catenin and Wnt10b in the dorsal skin of mice were significantly increased after WECP treatments in a dose-dependent manner. Simultaneously, we also detected the transcriptional expression of some other factors in the Wnt/β-Catenin signaling pathway. The results showed that the expression of Wnt10b, Wnt5a, β-Catenin, LEF1, IGF1, and VEGF, were significantly stimulated by WECP treatments (Figure 5C). These results suggested that WECP promotes the accumulation of β-Catenin and the expression of some downstream factors to promote hair growth of DPCs by activating Akt/GSK3β phosphorylation.

### 3.8 WECP reduced apoptosis and promoted HF cells proliferation

**FIGURE 6:**
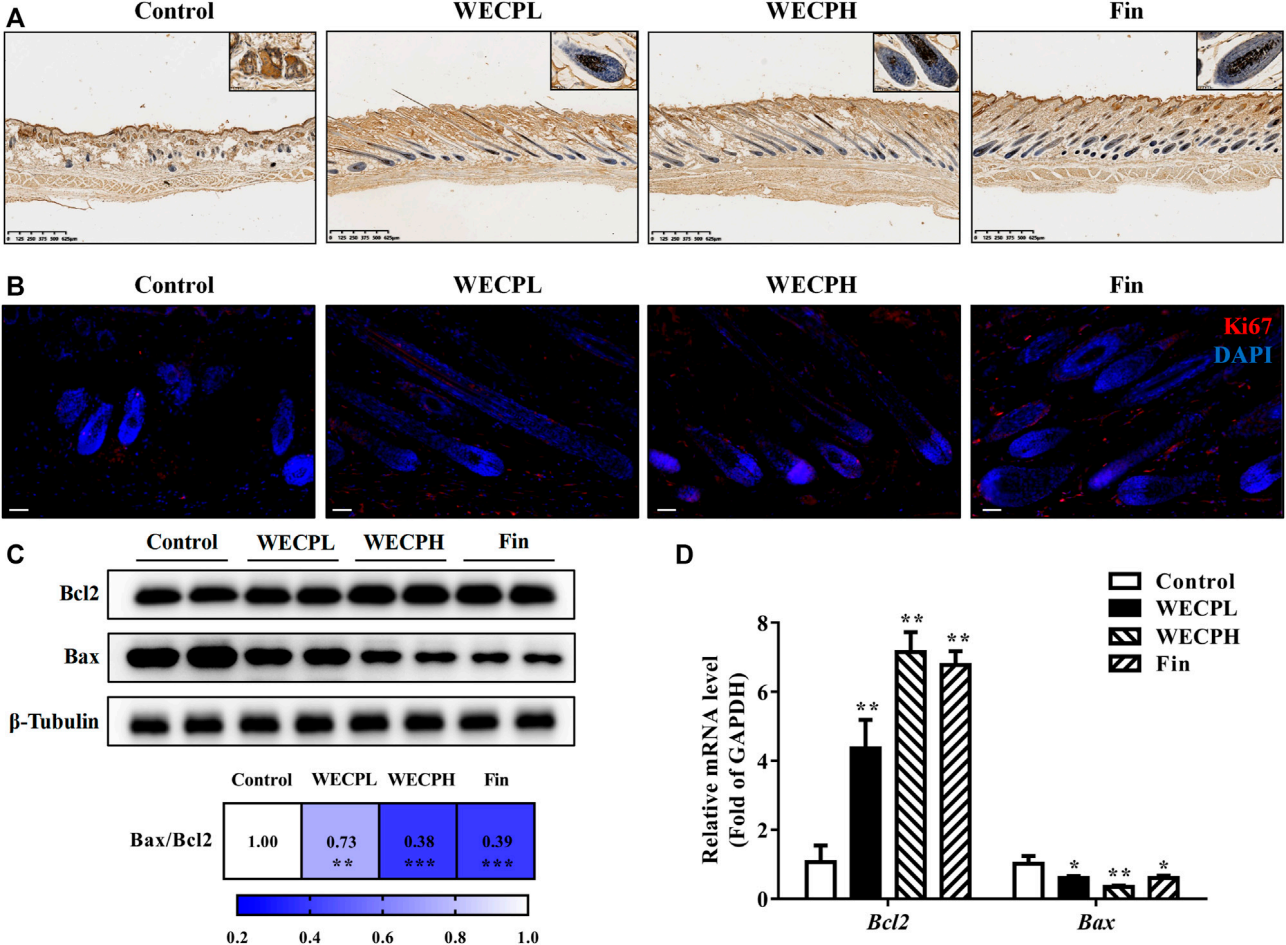
WECP attenuates apoptosis and promoted HF cells proliferationin in denuded mouse skin. **(A)** Representative sections showing TUNEL staining of hair follicles in mouse skin. Scale bars = 625 μm. **(B)** Representative sections showing Ki67 staining of hair follicles in mouse skin. Scale bars = 50 μm. Effects of WECP and finasteride treatments on **(C)** translational and **(D)** transcriptional expression of Bcl2 and Bax in mouse skin. Data are presented as means ± SD of three independent replicates. *p < 0.05, **p < 0.01, ***p < 0.001 vs. control group.

We performed TUNEL staining and ki67 immunofluorescence detection on mouse dorsal skin to assess whether WECP reduced apoptosis and promoted HF cells proliferation. There were significantly fewer apoptotic cells (TUNEL staining) in the dorsal skin and especially the hair bulbs of mice treated with WECP and Fin than there were in the dorsal skin of the Control mice (Figure 6A). On the contrary, a higher ki67 fluorescence intensity was observed in dorsal skin of the WECP and Fin group compared to that of the control (Figure 6B). Furthermore, we detected dermal Bcl2 and Bax expression by western blotting and RT-qPCR. The Bax/Bcl2 ratio was decreased to a greater extent in the mice treated with high WECP doses compared to those treated with low WECP doses (Figures 6C, D). These findings were consistent with those of the TUNEL staining assay. Therefore, WECP may reduce apoptosis and promote HF cells proliferation in mouse dorsal skin.

Inhibition of Akt signaling abolished the proliferative and migration-enhancing effects of WECP on DPCs.

**FIGURE 7:**
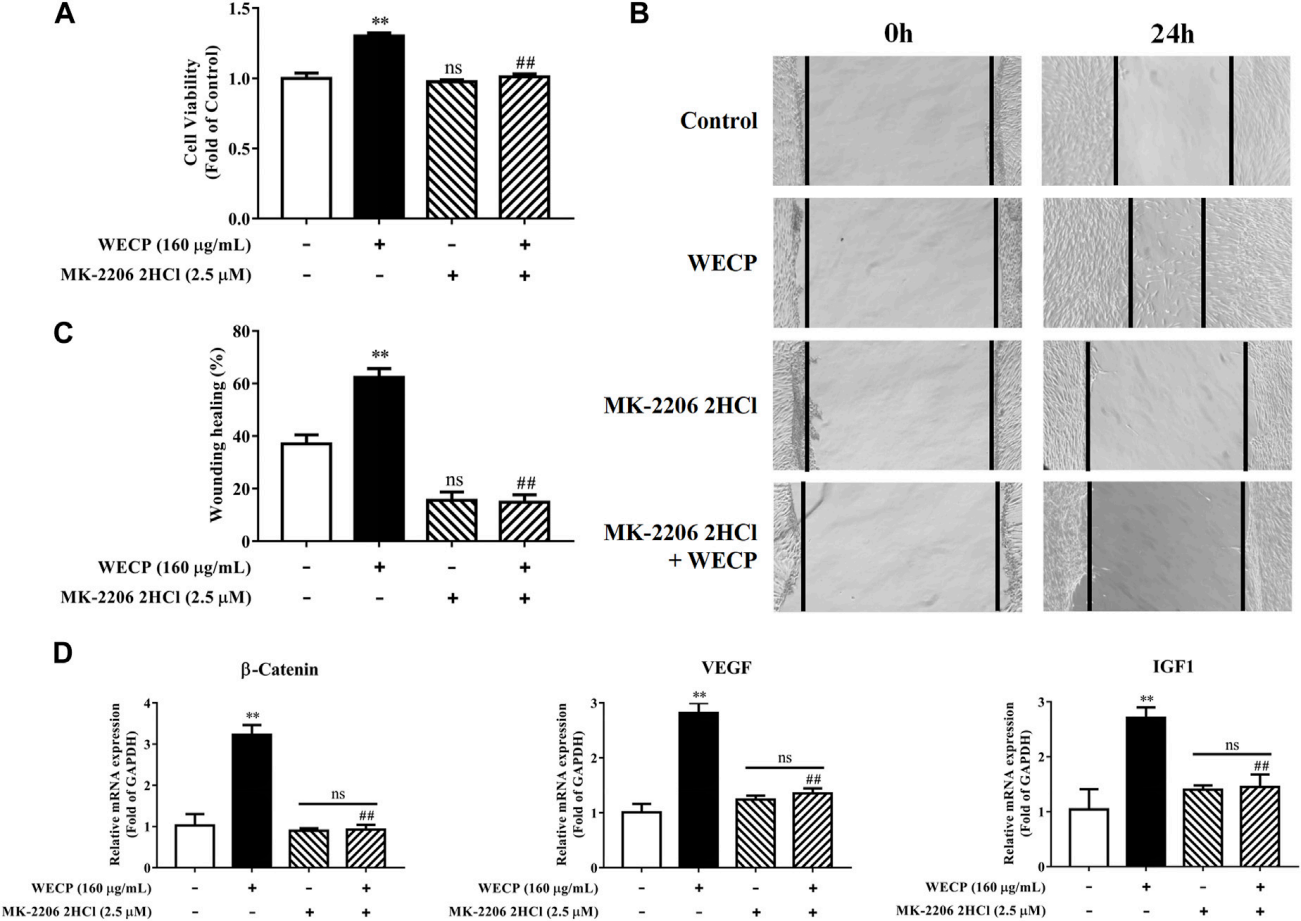
Abolition of the promoting effect of WECP on DPC proliferation and migration through Akt inhibition. **(A–D)** DPCs were treated with WECP (160 μg/mL) for 24 h. Akt inhibitor MK-2206 2HCl was added 1 h before WECP treatment. **(A)** Cell proliferation was measured by CCK-8 assay. **(B)** Microscopic images of scratched areas were captured. Lines indicate migrating cell edges. **(C)** DPC migration was quantitatively analyzed and shown as bar graph. **(D)** Transcriptional expression of β-Catenin, IGF1, and VEGF were detected in DPCs by RT-PCR. Data are presented as means ± SD of three independent experiments. **p < 0.01 vs control; ##*p* < 0.01 vs WECP group. Note: NS, not significant.

We then used the Akt inhibitor MK-2206 2HCl to validate the stimulatory effects of WECP on DPCs proliferation and migration. MK-2206 2HCl counteracted WECP-mediated improvement of DPCs proliferation (Figure 7A). Similar results were obtained for the wound healing assays. WECP failed to promote DPC migration in the presence of MK-2206 2HCl (Figures 7B, C). We also found that MK-2206 2HCl abolished the WECP-triggered transcriptional expression of β-Catenin, IGF1, and VEGF (Figure 7D). The preceding results demonstrated that WECP promotes DPCs proliferation and migration by activating the Akt signaling pathway.

## 4 Discussion

Hair loss may be induced by genetic factors, chemotherapy, radiation therapy, hormone imbalances, certain infections, and nutrient deficiencies. Although hair loss has no serious impact on overall or general health, the demand for safe, efficacious hair loss treatment non-etheless continues to grow. Minoxidil and finasteride are widely prescribed clinical drugs approved by the United States Food and Drug Administration. However, both drugs are associated with side effects such as scalp dryness, skin irritation, erectile dysfunction, and testicular pain (Ruksiriwanich et al., 2022). Traditional Chinese medicine effectively alleviates the symptoms of complex diseases in a multi-target, multi-component manner. The Compendium of Materia Medica (Ben Cao Gang mu in Chinese) reported that *Platycladus orientalis* (L.) Franco needles could promote hair growth. However, there are limited pharmacological or phytochemical reports available based on this botanical drug. Here, we observed that WECP could promote hair growth in mice (Figure 1), stimulate DPCs proliferation and migration, and accelerate the DPCs cell cycle *in vitro* (Figure 2). We elucidated the phytochemical character of WECP (Supplementary Table S1). We discovered that Akt might be the critical target of WECP with network analysis (Figure 3). This result helped us designing *in vitro* and *in vivo* experiments. At the molecular level, WECP mediates β-Catenin accumulation by promoting Akt and GSK3β phosphorylation (Figure 4). As a result of which, WECP upregulated LEF1, VEGF, and IGF1 in the model mice and DPCs (Figure 5). We also observed that WECP reduced apoptosis and promoted HF cells proliferation (Figure 6). Hence, WECP might have general anti-apoptotic efficacy. Finally, we applied an Akt inhibitor, MK-2206 2HCl, to verify the regulative activity of WECP. We found that MK-2206 2HCl could abolish the proliferation and migration-promoting effects of WECP on DPCs. As anticipated, MK-2206 2HCl also inhibited the stimulatory effect of WECP on the expression of β-Catenin, VEGF, and IGF1 (Figure 7).

DPCs play an important role in hair growth and might regulate the hair cycle (Kim et al., 2020). Most individuals with alopecia are deficient with DPCs. When the hair cycle switches from telogen to anagen phase, a self-cell division within the dermal papilla and cell influx from the dermal sheath co-occur (Pantelireis and Higgins, 2018). As a result, the number of DPCs in the dermal papilla is restored to the same level as that of the prior growth phase. Therefore, DPCs proliferation indicates the efficacy of hair growth treatments. Cell proliferation is closely associated with cell cycle progression. Cyclin D1 is a key protein in cell proliferation and initiates DNA synthesis (Kang et al., 2018). Cyclin-dependent kinases such as CDK2 and CDK4 are activated by cyclins and positively coordinate cell cycle progression along with them. Our results showed that WECP stimulated cell proliferation (Figure 2A) by promoting the cell cycle (Figures 2B, C) and upregulating cyclin D1, CDK2, and CDK4 (Figure 2D). WECP regulated apoptosis-related gene activity in mouse dorsal skin (Figures 6B, C). Thus, WECP might be implicated in DPC apoptosis. Notably, cell migration is a key element in HF development. Moreover, WECP could unambiguously promote DPC migration (Figure 2E).

Network analysis is an emerging interdisciplinary approach that integrates systems biology and bioinformatics. It holistically and systematically shows the relationship among drugs, targets, and diseases and visually represents drug-target interaction networks (Zhao et al., 2019). Our study disclosed that Akt might be a critical WECP target (Figure 3). It has been reported that Akt regulates not only cell proliferation by inhibiting apoptosis (Datta et al., 1997), but also cell metabolism, proliferation, and reprogramming (Tang et al., 2014). Bai et al. (2017) showed that increased Akt phosphorylation inhibited P21, upregulated cyclin D1, and promoted HFMSC transition from G1 to S. These published results encouraged us to demonstrate the regulative capability of WECP on Akt. Recent studies have shown that Akt kinase inhibits GSK3β through phosphorylation and promotes cell cycle progression (McCubrey et al., 2017; Kang et al., 2018). GSK3β regulates the Wnt canonical signaling pathway, stabilizes β-Catenin, and inhibits cyclin D1 degradation (Zheng et al., 2017). The Wnt/β-Catenin signaling pathway plays crucial roles in HFs development, including growth cycle restart and maintenance and HF cells proliferation and differentiation (Ito et al., 2007; Ouji et al., 2008). β-Catenin ablation prevented HFs formation in the embryonic epidermis. Forced expression of constitutively activated epidermal β-Catenin expanded HFs fate during development (Enshell-Seijffers et al., 2010). Stabilized β-Catenin interacted with TCF/LEF TFs and transactivated cell growth factors associated with hair regrowth (Akiyama, 2000). Moreover, some previous studies reported that ingredients, such as rutin (Luo et al., 2022), isoquercitrin (Zhu et al., 2016), and myricitrin (Zhang et al., 2016) could regulate Akt. Additionally, rutin could also prevent apoptosis of DPCs (Carelli et al., 2012). Prevention and treatment of alopecia areata by quercetin has been studied using a C3H/HeJ mouse model (Wikramanayake et al., 2012). Therefore, we primarily focused on Akt/GSK3β/β-Catenin signaling pathway in the following study.

We found that WECP inhibits GSK3β by promoting Akt phosphorylation at the serine nine residue (Figure 4A). Our results also demonstrated that WECP promoted the transcription and translation of β-Catenin and Wnt10b, induced DPCs growth, and upregulated Wnt5a and LEF1 in mouse dorsal skin (Figures 4, 5). The Wnt/β-Catenin signaling pathway may also mediate the secretion of various growth factors in DPCs (Kim et al., 2020). IGF1 is a structural insulin homolog expressed in the MSCs of the dermal papilla and dermis, which can stimulate HFs development (Yu et al., 2020). VEGF induces vascularization around the HFs to promote hair growth (Zhang et al., 2020). Here, we found that WECP upregulated IGF1 and VEGF. This suggested a critical role of the Wnt/β-Catenin signaling pathway in WECP-mediated hair growth. To confirm prior results, we treated DPCs with the Akt inhibitor MK-2206 2HCl before the WECP application. MK-2206 2HCl abolished the proliferation- and migration-promoting effects of WECP and restored β-Catenin, IGF1, and VEGF expression (Figure 7). Interestingly, we found that the DPCs proliferation and migration improving capability of WECP (having complex chemical components) could be inhibited with one chemical molecule MK-2206 2HCl. This suggested that Akt is not only acts as the key factor, and but also acts the initiator of the WHCP-regulated pathway. However, this hypothesis still needs to be verified using MK-2206 2HCl treated or gene-modified hair loss model mice.

**FIGURE 8:**
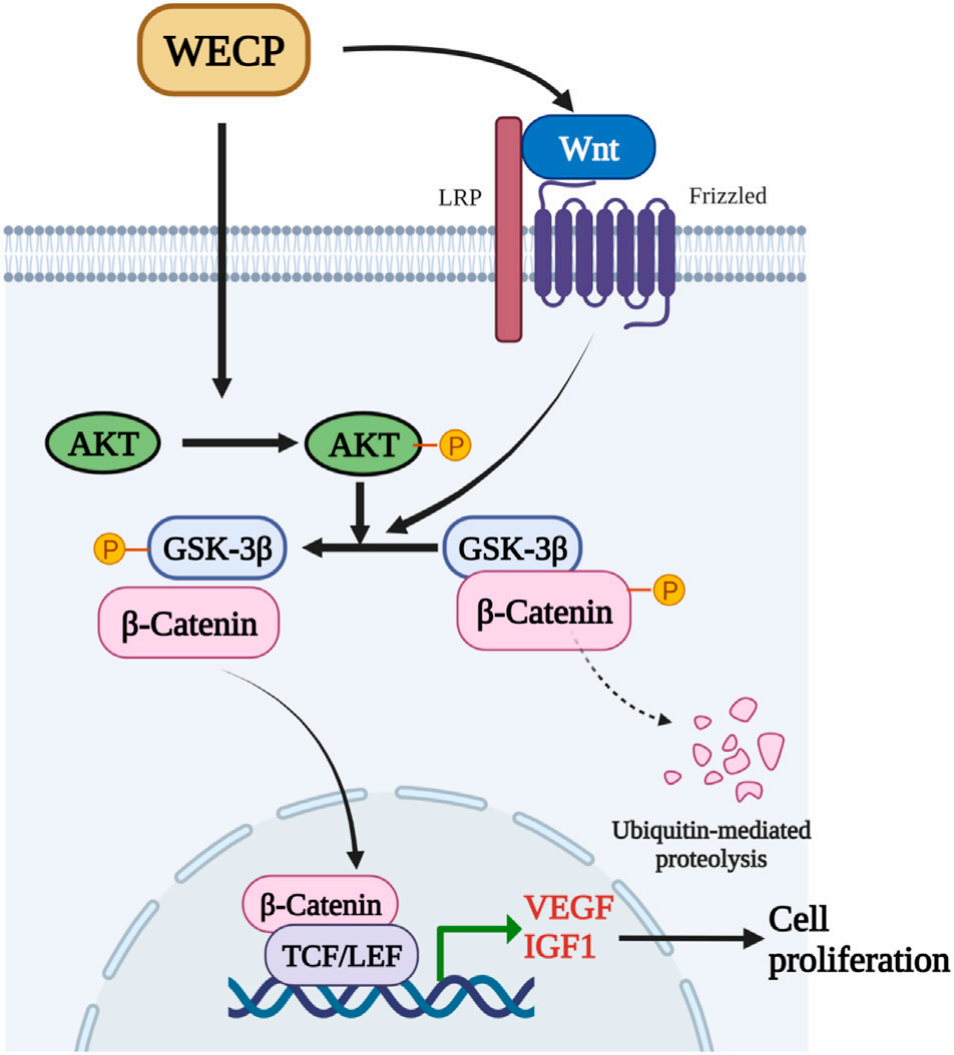
WECP mediates β-Catenin accumulation and transcription by promoting Akt and GSK3β phosphorylation. β-Catenin translocates to the nucleus where it enhances LEF1 factor transactivation and upregulates VEGF and IGF1.

The results of the present study suggested that the hair growth improvement capability of WECP in mice was mediated by DPCs proliferation and migration. WECP attenuated GSK3β and upregulated β-Catenin by activating Akt (Figure 8). Since the mixture of the ingredients in WECP tend to be quite complex, future research should focus on the effect of these ingredients in WECP on hair growth and hair cell cycle regulation. As we understand, novel therapies against hair loss are under development. We hope to identify some beneficial compounds or therapeutic combinations to combat hair loss.

## Data Availability

The datasets presented in this study can be found in online repositories. The names of the repository/repositories and accession number(s) can be found in the article/Supplementary Material.

## Abbreviations

CP, Cacumen Platycladi; WECP, Water extract of Cacumen Platycladi; HFs, Hair follicles; DPCs, Dermal papilla cells; CDK2, Cyclin-dependent kinase 2; CDK4 Cyclin-dependent kinase 4; GSK3β, Glycogen synthase kinase-3-beta; LEF1, Lymphoid enhancer-binding factor 1; VEGF, Vascular endothelial growth factor; IGF1, Insulin-like growth factor 1; Wnt, Wingless-INT; Fin, Finasteride; UPLC-Q/TOF-MS, Ultrahigh-performance liquid chromatography-quadrupole/time-of-flight Mass spectrometry system; OB, Oral bioavailability; DL, Drug-likeness; PPI, Protein-protein interaction; GO, Gene ontology; KEGG, Kyoto Encyclopedia of Genes and Genomes; PI, Propidium iodide; PBS, Phosphate-buffered saline; RT-qPCR, Real-Time Quantitative Polymerase Chain Reaction; WECPL, Low-dose WECP; WECPH, High-dose WECP; H&E, Hematoxylin and eosin; SD, Standard deviation; BP, Biological process; CC, Cellular component; MF, Molecular function; HFMSCs, Hair follicle mesenchymal stem cells.
