#  Corrigendum: Water extract of cacumen platycladi promotes hair growth through the Akt/GSK3β/β-catenin signaling pathway

**DOI:** 10.3389/fphar.2023.1200103

**Published:** 2023-05-26

**Authors:** Hangjie Fu, Wenxia Li, Zhiwei Weng, Zhiguang Huang, Jinyuan Liu, Qingqing Mao, Bin Ding

**Affiliations:** ^1^ College of Life Science, Zhejiang Chinese Medical University, Hangzhou, China; ^2^ Academy of Chinese Medical Science, Zhejiang Chinese Medical University, Hangzhou, China; ^3^ The Fourth School of Clinical Medicine, Zhejiang Chinese Medical University, Hangzhou, China

**Keywords:** alopecia, cacumen platycladi, hair follicles, dermal papilla cells, Akt, GSK3β

In the published article, there was an error in Figure 7 as published. In Figure 7B, the images of the MK2206 2HCl and MK2206 2HCI + WECP groups were repeatedly pasted at 0 h. However, we did not find this error in the original data, only a repeated pasting error occurred during the image arrangement process. Therefore, we made image corrections based on the original data**.** The corrected Figure 7 and its caption appear below.

**FIGURE 7:**
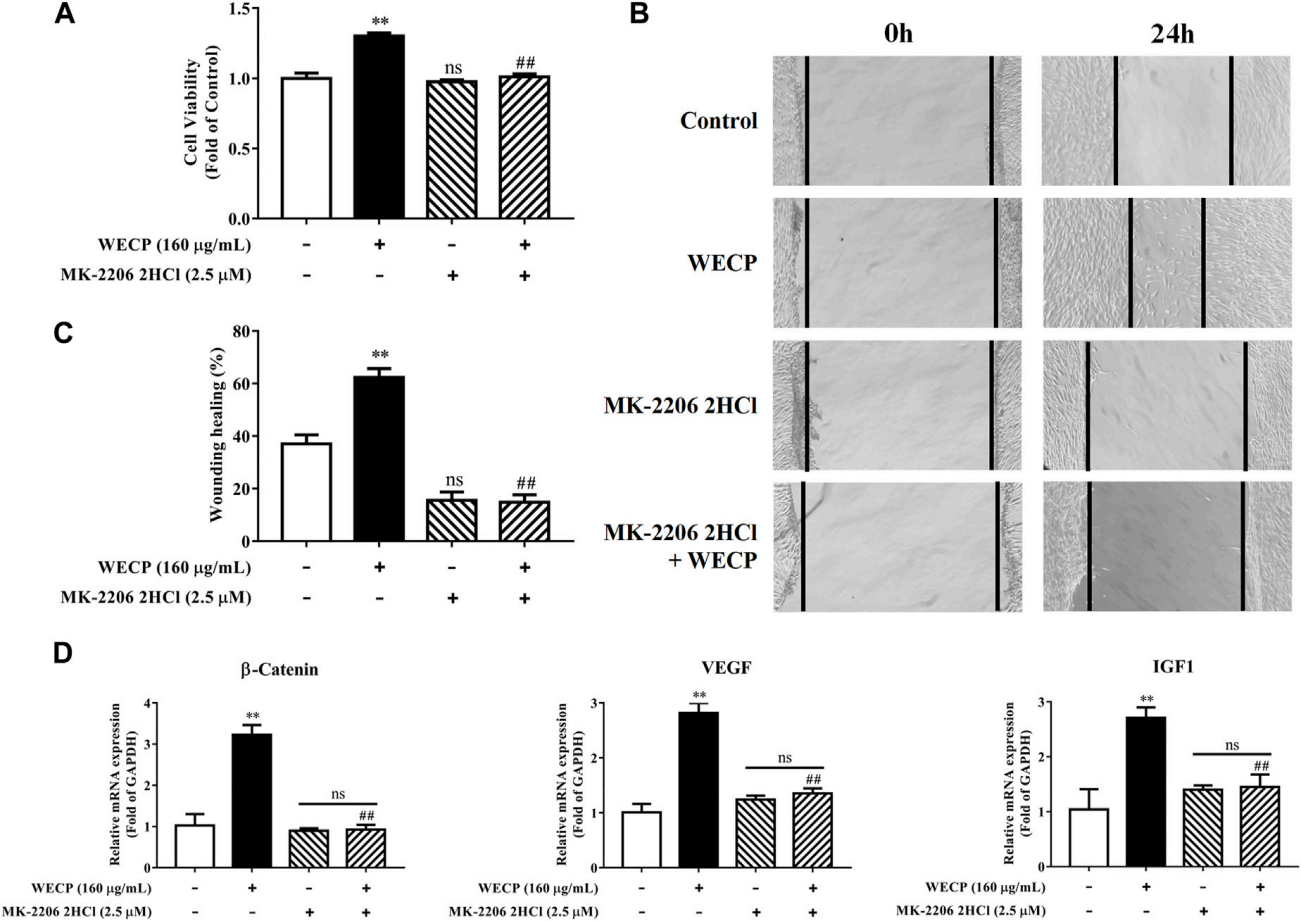
Abolition of the promoting effect of WECP on DPC proliferation and migration through Akt inhibition. **(A–D)** DPCs were treated with WECP (160 μg/mL) for 24 h. Akt inhibitor MK-2206 2HCl was added 1 h before WECP treatment. **(A)** Cell proliferation was measured by CCK-8 assay. **(B)** Microscopic images of scratched areas were captured. Lines indicate migrating cell edges. **(C)** DPC migration was quantitatively analyzed and shown as bar graph. **(D)** Transcriptional expression of β-Catenin, IGF1, and VEGF were detected in DPCs by RT-PCR. Data are presented as means ± SD of three independent experiments. **p < 0.01 vs control; ##*p* < 0.01 vs WECP group. Note: NS, not significant.

The authors apologize for this error and state that this does not change the scientific conclusions of the article in any way. The original article has been updated.

