# Supplementary material for: Water extract of cacumen platycladi promotes hair growth through the Akt/GSK3β/β-catenin signaling pathway
Source: Front Pharmacol. 2023 Feb 20;14:1038039. doi: 10.3389/fphar.2023.1038039 (PMC9986263; doi:10.3389/fphar.2023.1038039)
Supplement: Supplementary file 1 [file DataSheet1.docx]

Supplementary Material

# Supplementary Data

Supplementary Material should be uploaded separately on submission. Please include any supplementary data, figures and/or tables. All supplementary files are deposited to FigShare for permanent storage and receive a DOI.

Supplementary material is not typeset so please ensure that all information is clearly presented, the appropriate caption is included in the file and not in the manuscript, and that the style conforms to the rest of the article. To avoid discrepancies between the published article and the supplementary material, please do not add the title, author list, affiliations or correspondence in the supplementary files.

# Supplementary Figures and Tables

For more information on Supplementary Material and for details on the different file types accepted, please see [here](http://home.frontiersin.org/about/author-guidelines#SupplementaryMaterial). Figures, tables, and images will be published under a Creative Commons CC-BY licence and permission must be obtained for use of copyrighted material from other sources (including re-published/adapted/modified/partial figures and images from the internet). It is the responsibility of the authors to acquire the licenses, to follow any citation instructions requested by third-party rights holders, and cover any supplementary charges.

## Supplementary Figures


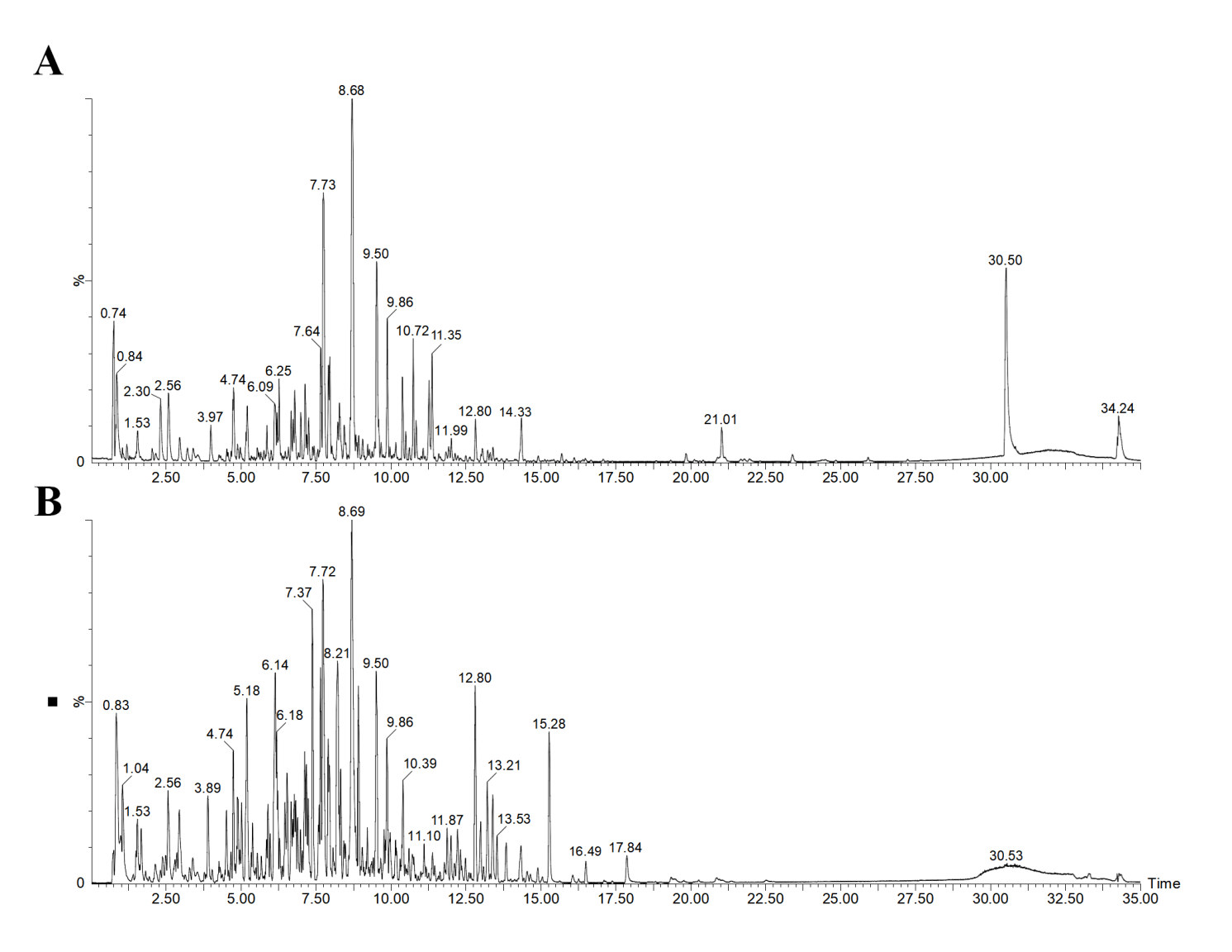


**Supplementary Figure 1.** Chemical composition analysis of Water extract of Cacumen Platycladi by UPLC-TOF-MS under negative and positive ion mode. (A) negative ion mode. (B) positive ion mode.

**Table S1-A** Chemical composition analysis of Water extract of Cacumen Platycladi by UPLC-TOF/MS under positive ion mode.

| **Compound** | **Retention time/min** | **Name** | **Chemical Formula** | **Molecular Mass/Da** | **m/z Observation** | **δ/mDa** | **Adduct** |
| --- | --- | --- | --- | --- | --- | --- | --- |
| **1** | **5.15** | **Hinokinin** | **C20H18O6** | **354.11034** | **377.0975** | **-2** | **+Na** |
| **2** | **6.1** | **α** － Cadinol | **C15H26O** | **222.19837** | **245.1854** | **-2.2** | **+Na** |
| **3** | **6.14** | **p-cymene** | **C10H14** | **134.10955** | **135.1166** | **-0.2** | **+H** |
| **4** | **6.18** | **Rutin** | **C27H30O16** | **610.15338** | **611.1604** | **-0.3** | **+H** |
| **5** | **7.72** | **Isoquercitrin** | **C21H20O12** | **464.09548** | **465.1025** | **-0.3** | **+H, +Na, +K** |
| **6** | **7.94** | **Quercetin** | **C15H10O7** | **302.04265** | **303.0499** | **-0.1** | **+H** |
| **7** | **8.01** | **Myricetin** | **C15H10O8** | **318.03757** | **319.0447** | **-0.1** | **+H** |
| **8** | **8.27** | **Myricitrin** | **C21H20O12** | **464.09548** | **465.1023** | **-0.4** | **+H, +Na** |
| **9** | **8.91** | **Glabridin** | **C20H20O4** | **324.13616** | **325.1413** | **-2.1** | **+H** |
| **10** | **11.11** | **Cuparene** | **C15H22** | **202.17215** | **203.1792** | **-0.3** | **+H** |
| **11** | **12** | **Licoflavanone** | **C20H20O5** | **340.13107** | **341.1383** | **0** | **+H** |
| **12** | **12.8** | **3-hexen-1-01**，benzoate | **C13H16O2** | **204.11503** | **205.1222** | **-0.1** | **+H** |
| **13** | **15.68** | **ferruginol** | **C20H30O** | **286.22967** | **287.2362** | **-0.7** | **+H** |
| **14** | **30.68** | **DNOP** | **C24H38O4** | **390.27701** | **413.2659** | **-0.3** | **+Na, +H, +K** |

**Table S1-B** Chemical composition analysis of Water extract of Cacumen Platycladi by UPLC-TOF/MS under negative ion mode.

| **Compound** | **Retention time/min** | **Name** | **Chemical Formula** | **Molecular Mass/Da** | **m/z Observation** | **δ/mDa** | **Adduct** |
| --- | --- | --- | --- | --- | --- | --- | --- |
| **1** | **7.06** | **Isoquercitrin** | **C21H20O12** | **464.09548** | **463.0879** | **-0.3** | **-H** |
| **2** | **8.01** | **Myricetin** | **C15H10O8** | **318.03757** | **317.0297** | **-0.5** | **-H** |
| **3** | **8.77** | **dextrose** | **C6H12O6** | **180.06339** | **179.055** | **-1.1** | **-H** |
| **4** | **8.56** | **Rutin** | **C27H30O16** | **610.15338** | **609.1449** | **-1.2** | **-H** |
| **5** | **9.04** | **Quercetin** | **C15H10O7** | **302.04265** | **301.0348** | **-0.5** | **-H** |
| **6** | **13.6** | **hinokiflavone** | **C30H18O10** | **538.09** | **537.0826** | **-0.1** | **-H** |

**Table S2** Basic information of WECP ingredients.

| **Compound** | **Name** | **OB (%)** | **DL** |
| --- | --- | --- | --- |
| **1** | **Glabridin** | **53.25** | **0.47** |
| **2** | **ferruginol** | **36.07** | **0.25** |
| **3** | **[Hinokinin](https://tcmsp-e.com/molecule.php?qn=2005)** | **56.5** | **0.64** |
| **4** | **[DNOP](https://tcmsp-e.com/molecule.php?qn=2032)** | **40.59** | **0.4** |
| **5** | **[Quercetin](https://tcmsp-e.com/molecule.php?qn=98)** | **46.43** | **0.28** |
| **6** | **[Isopimaric acid](https://tcmsp-e.com/molecule.php?qn=2039)** | **36.2** | **0.28** |
| **7** | **[beta-sitosterol](https://tcmsp-e.com/molecule.php?qn=358)** | **36.91** | **0.75** |
| **8** | **[Kaempferol](https://tcmsp-e.com/molecule.php?qn=422)** | **41.88** | **0.24** |
| **9** | **Deoxypicropodophyllotoxin** | **52.7** | **0.83** |

**Table S3** Basic information of Hair loss Targets in GeneCards and DisGeNET.

| **GeneCards** | | **DisGeNET** | |
| --- | --- | --- | --- |
| **Gene Symbol** | **Relevance score** | **Gene** | **Score_gda** |
| **DSP** | **148.0626221** | **VDR** | **0.5** |
| **GJB2** | **108.4439621** | **SRD5A2** | **0.43** |
| **MYO7A** | **80.65213013** | **HR** | **0.43** |
| **CDH23** | **78.8959198** | **LSS** | **0.42** |
| **GJB6** | **70.43185425** | **HTRA1** | **0.41** |
| **KRT74** | **65.30822754** | **EDNRA** | **0.4** |
| **EDAR** | **64.86467743** | **AR** | **0.39** |
| **MYO15A** | **61.06599808** | **MTHFR** | **0.32** |
| **OTOF** | **59.60652924** | **ABCC2** | **0.31** |
| **TYR** | **54.99828339** | **ZDHHC13** | **0.31** |
| **LPAR6** | **54.48653793** | **CRH** | **0.31** |
| **LIPH** | **54.20776367** | **BRD4** | **0.3** |
| **TECTA** | **53.69771194** | **ODC1** | **0.3** |
| **LOXHD1** | **51.68637848** | **SUPV3L1** | **0.3** |
| **KRT25** | **51.52095413** | **ZFP36** | **0.3** |
| **SLC26A4** | **51.27930832** | **PARP1** | **0.3** |
| **EDARADD** | **51.21496582** | **TNFRSF10A** | **0.3** |
| **KRT83** | **50.56837082** | **PRKAR1A** | **0.3** |
| **MC1R** | **50.35260391** | **FAR2** | **0.21** |
| **WFS1** | **50.05747604** | **AIRE** | **0.18** |
| **DIAPH1** | **49.53105164** | **NUDT15** | **0.16** |
| **RMRP** | **49.29093933** | **HLA-DRB1** | **0.16** |
| **MYO6** | **49.12971115** | **PTPN22** | **0.15** |
| **HOXC13** | **49.06990433** | **IL2RA** | **0.15** |
| **KRT86** | **47.05377197** | **LIPH** | **0.14** |
| **KRT85** | **46.87740326** | **FOXN1** | **0.14** |
| **KCNQ4** | **46.73229218** | **LPAR6** | **0.13** |
| **MYH9** | **46.51598358** | **TRPV3** | **0.12** |
| **DSG4** | **45.34287262** | **HLA-DQB1** | **0.12** |
| **ATP7A** | **45.32197952** | **FOXP3** | **0.12** |
| **KRT81** | **44.77950287** | **MBTPS2** | **0.12** |
| **ACTG1** | **44.35727692** | **HLA-DQA1** | **0.12** |
| **TMC1** | **44.10379791** | **TGM1** | **0.11** |
| **TBC1D24** | **43.83423615** | **ZMPSTE24** | **0.11** |
| **MITF** | **43.79360962** | **KRT74** | **0.11** |
| **KRT71** | **43.70037079** | **AHSG** | **0.11** |
| **FGFR3** | **43.45436096** | **TRPS1** | **0.11** |
| **USH1C** | **42.68574905** | **GJB2** | **0.11** |
| **MYO3A** | **42.26832199** | **DSP** | **0.11** |
| **TP53** | **42.23542404** | **DSG4** | **0.11** |
| **RANBP2** | **41.62532806** | **BCL2** | **0.11** |
| **TP63** | **41.57452393** | **TARDBP** | **0.11** |
| **GJA1** | **41.51570511** | **SPP1** | **0.11** |
| **TGM3** | **40.93160629** | **EBP** | **0.11** |
| **KRAS** | **39.90120697** | **DCAF17** | **0.11** |
| **TBCEL-TECTA** | **39.83549118** | **RBM28** | **0.11** |
| **PADI3** | **39.82524109** | **ITGB6** | **0.11** |
| **PCDH15** | **39.23581696** | **CTLA4** | **0.11** |
| **KRT75** | **39.21339798** | **NOP10** | **0.1** |
| **SHOC2** | **38.93154144** | **HAUS7** | **0.1** |
| **KITLG** | **38.64414215** | **PDGFA** | **0.1** |
| **MYH14** | **37.74305344** | **PEX14** | **0.1** |
| **USH2A** | **37.67919922** | **RTEL1** | **0.1** |
| **DSC2** | **37.63950348** | **SLC39A4** | **0.1** |
| **TSPEAR** | **37.57915497** | **PEX7** | **0.1** |
| **WNT10A** | **37.42440796** | **TTC27** | **0.1** |
| **TMPRSS3** | **37.26536942** | **PIK3R1** | **0.1** |
| **GJB3** | **37.10015488** | **LGR4** | **0.1** |
| **OCA2** | **37.09369659** | **WRAP53** | **0.1** |
| **TCHH** | **36.93971634** | **C2orf42** | **0.1** |
| **CTNNB1** | **36.82029724** | **CASZ1** | **0.1** |
| **WHRN** | **36.40778351** | **DLL4** | **0.1** |
| **HR** | **36.16649628** | **ATR** | **0.1** |
| **EYA4** | **35.93272781** | **RIN2** | **0.1** |
| **POMC** | **35.93126297** | **SOX18** | **0.1** |
| **COCH** | **35.77419281** | **TSPEAR** | **0.1** |
| **LMNA** | **35.70572281** | **TSPEAR-AS1** | **0.1** |
| **SPATA5** | **35.29184723** | **PLEC** | **0.1** |
| **ERCC2** | **35.16615295** | **PKP1** | **0.1** |
| **PRPS1** | **34.73166656** | **SLC29A3** | **0.1** |
| **CDH3** | **33.86106873** | **ABHD5** | **0.1** |
| **COL11A2** | **33.43976593** | **MEMO1** | **0.1** |
| **JUP** | **33.39715195** | **KRT85** | **0.1** |
| **LHFPL5** | **33.07930374** | **KRT83** | **0.1** |
| **SDHD** | **33.0465889** | **KRT81** | **0.1** |
| **SOX10** | **32.98223114** | **KRT17** | **0.1** |
| **PAX3** | **32.69828033** | **KRT16** | **0.1** |
| **RB1** | **32.5980072** | **KRT6B** | **0.1** |
| **PTEN** | **32.19886398** | **KRT6A** | **0.1** |
| **BRAF** | **31.86478424** | **KRAS** | **0.1** |
| **PPP1CB** | **31.62271118** | **FAM111B** | **0.1** |
| **POU3F4** | **31.30604935** | **JUP** | **0.1** |
| **TMIE** | **31.21197319** | **IRF4** | **0.1** |
| **PJVK** | **31.19917297** | **IRAK1** | **0.1** |
| **HRAS** | **31.11450195** | **EIF3E** | **0.1** |
| **TRIOBP** | **31.02218437** | **IL7R** | **0.1** |
| **CDSN** | **30.91500664** | **IL2RG** | **0.1** |
| **EDA** | **30.74252129** | **RBPJ** | **0.1** |
| **BCS1L** | **30.67159843** | **NANOGP9** | **0.1** |
| **SLC26A5** | **30.0999794** | **KRT86** | **0.1** |
| **STRC** | **30.02453613** | **LAMA3** | **0.1** |
| **PIK3CA** | **29.87282944** | **LAMB3** | **0.1** |
| **EGFR** | **29.62213135** | **PAX3** | **0.1** |
| **TYRP1** | **29.33287048** | **PARN** | **0.1** |
| **KANK2** | **29.28819847** | **OPHN1** | **0.1** |
| **CEP78** | **29.17067146** | **ROR2** | **0.1** |
| **CEACAM16** | **29.16048622** | **NSF** | **0.1** |
| **ATP6V1B1** | **28.56162643** | **NRAS** | **0.1** |
| **KRT17** | **28.35015106** | **NOTCH1** | **0.1** |
| **KARS1** | **28.28294754** | **MSN** | **0.1** |
| **HERC2** | **28.24935913** | **MMP1** | **0.1** |
| **MT-TS1** | **28.07202148** | **MKLN1** | **0.1** |
| **NRAS** | **27.69133759** | **MGP** | **0.1** |
| **KRT14** | **27.60409164** | **MAPT** | **0.1** |
| **SLC45A2** | **27.58009338** | **TEX41** | **0.1** |
| **DCDC2** | **27.46352005** | **LMNA** | **0.1** |
| **CLDN14** | **27.39363861** | **LIG4** | **0.1** |
| **MPLKIP** | **27.34010506** | **THADA** | **0.1** |
| **AR** | **27.31006622** | **LAMC2** | **0.1** |
| **SMARCA2** | **27.29040527** | **NIPAL4** | **0.1** |
| **ERCC6** | **27.27806091** | **C1QA** | **0.1** |
| **TERT** | **27.18792725** | **XPC** | **0.1** |
| **ATP1A3** | **27.06541824** | **SLC30A2** | **0.1** |
| **KCNJ10** | **26.79642296** | **ALMS1** | **0.1** |
| **POU4F3** | **26.77427864** | **EPS8L3** | **0.1** |
| **COL11A1** | **26.70806122** | **USB1** | **0.1** |
| **EDNRB** | **26.64599991** | **CTC1** | **0.1** |
| **KDF1** | **26.18407249** | **WNT10A** | **0.1** |
| **ERCC3** | **26.17424011** | **COL18A1** | **0.1** |
| **MT-TL1** | **26.12144852** | **XPA** | **0.1** |
| **SLC17A8** | **26.12114334** | **XDH** | **0.1** |
| **FOXE1** | **26.11943817** | **TNFRSF1B** | **0.1** |
| **NF1** | **26.09361839** | **C1R** | **0.1** |
| **ESPN** | **26.02741241** | **HULC** | **0.1** |
| **IGF1** | **25.96347809** | **TWIST1** | **0.1** |
| **GSDME** | **25.60376358** | **UROD** | **0.1** |
| **CDH1** | **25.24276161** | **UROS** | **0.1** |
| **CDKN2A** | **25.15135765** | **WNT5A** | **0.1** |
| **IL6** | **25.11885452** | **WNT6** | **0.1** |
| **PEX6** | **25.04936028** | **SSPN** | **0.1** |
| **ATM** | **25.04005051** | **SLC14A2** | **0.1** |
| **MT-ND1** | **24.9583149** | **LRMDA** | **0.1** |
| **F5** | **24.92706299** | **PRDM6** | **0.1** |
| **CARS1** | **24.82261086** | **CD28** | **0.1** |
| **CHD7** | **24.78511047** | **RECQL4** | **0.1** |
| **RDX** | **24.77371979** | **FADS2** | **0.1** |
| **ARID1B** | **24.72473145** | **FAM53B** | **0.1** |
| **PTPRQ** | **24.7203083** | **VGLL4** | **0.1** |
| **INS** | **24.69992828** | **HDAC9** | **0.1** |
| **ZMPSTE24** | **24.67077446** | **PLEKHM1** | **0.1** |
| **FOXN1** | **24.6617012** | **CLDN1** | **0.1** |
| **OPA1** | **24.58365631** | **ACVR1** | **0.1** |
| **MYO5A** | **24.51574707** | **CASR** | **0.1** |
| **BRCA1** | **24.45322037** | **DPY30** | **0.1** |
| **SMAD4** | **24.41213799** | **OFD1** | **0.1** |
| **CIB2** | **24.37726593** | **IKBKG** | **0.1** |
| **FGFR1** | **24.29849625** | **RUNX1** | **0.1** |
| **F2** | **24.20228386** | **TP63** | **0.1** |
| **TRPS1** | **24.10863686** | **RUNX3** | **0.1** |
| **RET** | **24.0789547** | **IQGAP1** | **0.1** |
| **FGFR2** | **23.90930939** | **ZC3H11A** | **0.1** |
| **EXOSC2** | **23.79283524** | **CHD7** | **0.1** |
| **CLDN14-AS1** | **23.78202629** | **DOCK6** | **0.1** |
| **KCNQ1** | **23.74581528** | **NECTIN1** | **0.1** |
| **SIX1** | **23.72182655** | **ZNF462** | **0.1** |
| **ABHD12** | **23.63874054** | **RAG1** | **0.1** |
| **KLHL24** | **23.48900032** | **RAG2** | **0.1** |
| **MT-RNR1** | **23.4602108** | **ALOXE3** | **0.1** |
| **MSX1** | **23.25697327** | **RMRP** | **0.1** |
| **DPH1** | **23.11211586** | **TRMT11** | **0.1** |
| **TRAF6** | **23.08506966** | **ARHGAP31** | **0.1** |
| **MT-CO1** | **22.94236946** | **BBX** | **0.1** |
| **USH1G** | **22.90951347** | **NHP2** | **0.1** |
| **ADGRV1** | **22.86264038** | **PRKACA** | **0.1** |
| **CLRN1** | **22.81450844** | **EXOC2** | **0.1** |
| **SLC12A2** | **22.75352859** | **PRKD1** | **0.1** |
| **HGF** | **22.73228645** | **BTNL2** | **0.1** |
| **AKT1** | **22.69411087** | **DUSP22** | **0.1** |
| **KIT** | **22.67003441** | **MRPS22** | **0.1** |
| **MAP2K1** | **22.65200806** | **ANTXR1** | **0.1** |
| **MECP2** | **22.57384491** | **ALX4** | **0.1** |
| **TNF** | **22.56219101** | **RORA** | **0.1** |
| **CCDC107** | **22.4629631** | **RPL21** | **0.1** |
| **DSC3** | **22.4589653** | **BRAF** | **0.1** |
| **PTPN11** | **22.37101746** | **STAT4** | **0.1** |
| **LSS** | **22.31223297** | **BTD** | **0.1** |
| **SOX18** | **22.25653839** | **TBX15** | **0.1** |
| **SMPX** | **21.93203735** | **TCF4** | **0.1** |
| **CDC14A** | **21.90577698** | **TCF12** | **0.1** |
| **AIFM1** | **21.89159203** | **BTK** | **0.1** |
| **IKBKG** | **21.85216141** | **TERC** | **0.1** |
| **SPINK5** | **21.81519127** | **SNRPE** | **0.1** |
| **KRT35** | **21.80103874** | **SMARCA2** | **0.1** |
| **MARVELD2** | **21.72583008** | **BCS1L** | **0.1** |
| **APOE** | **21.64457512** | **PERP** | **0.1** |
| **PPARG** | **21.6049099** | **MCCC2** | **0.1** |
| **SNAI2** | **21.59367752** | **NXN** | **0.1** |
| **EDA2R** | **21.57718468** | **DCLRE1C** | **0.1** |
| **ESRRB** | **21.57289505** | **ST3GAL4** | **0.1** |
| **RIPOR2** | **21.54942513** | **PORCN** | **0.1** |
| **OTOG** | **21.46640015** | **BMP2** | **0.1** |
| **SOX9** | **21.41368484** | **TERT** | **0.1** |
| **KRT31** | **21.41106033** | **RSPO2** | **0.1** |
| **SNRPE** | **21.39493561** | **SRRM1** | **0.1** |
| **LRTOMT** | **21.3696537** | **OFCC1** | **0.1** |
| **IFT140** | **21.33826828** | **C1orf127** | **0.1** |
| **ATP6V0A4** | **21.31713867** | **UBR1** | **0.1** |
| **SPATA5L1** | **21.29238319** | **LINC01995** | **0.1** |
| **ALMS1** | **21.19233704** | **LINC00311** | **0.1** |
| **EDN3** | **21.1356144** | **ARHGEF9** | **0.1** |
| **GIPC3** | **21.10083008** | **SPAG17** | **0.1** |
| **IARS2** | **21.07302666** | **MTX2** | **0.1** |
| **GRHL2** | **21.04448318** | **CERS3** | **0.1** |
| **TNC** | **20.99638367** | **TWIST2** | **0.1** |
| **RPL21** | **20.96585846** | **LINC01681** | **0.1** |
| **ITGB4** | **20.96331215** | **GJA1** | **0.1** |
| **DNMT1** | **20.89952469** | **LINC00670** | **0.1** |
| **ASIP** | **20.89735794** | **STUB1** | **0.1** |
| **CEP250** | **20.89580917** | **GNA11** | **0.1** |
| **CST6** | **20.74485779** | **WARS2** | **0.1** |
| **TOMT** | **20.70109558** | **DVL1** | **0.1** |
| **NOTCH1** | **20.6429863** | **DKK2** | **0.1** |
| **LARS2** | **20.62279129** | **DVL3** | **0.1** |
| **IL10** | **20.60831451** | **EBF1** | **0.1** |
| **FLG** | **20.52957153** | **GJB3** | **0.1** |
| **PDZD7** | **20.50850677** | **GJB4** | **0.1** |
| **LRRC56** | **20.46918297** | **ERCC2** | **0.1** |
| **DLX5** | **20.42980576** | **ERCC3** | **0.1** |
| **COL17A1** | **20.42856598** | **GJB6** | **0.1** |
| **EYA1** | **20.41305542** | **KLF8** | **0.1** |
| **PSAP** | **20.37696457** | **FZD2** | **0.1** |
| **TGFB1** | **20.36559486** | **ZHX3** | **0.1** |
| **IRF4** | **20.18268204** | **ZBTB38** | **0.1** |
| **POLG** | **20.16747475** | **KDSR** | **0.1** |
| **RAB27A** | **20.13587952** | **GSE1** | **0.1** |
| **GTF2H5** | **19.98703194** | **SASH1** | **0.1** |
| **APCDD1** | **19.98381042** | **FTL** | **0.1** |
| **NGF** | **19.96363258** | **EXOSC2** | **0.1** |
| **POGZ** | **19.94611359** | **SLITRK1** | **0.1** |
| **COL4A6** | **19.92915916** | **SETBP1** | **0.1** |
| **HEPHL1** | **19.9220562** | **FAF1** | **0.1** |
| **GRXCR1** | **19.8902626** | **PNPLA6** | **0.1** |
| **SLC29A3** | **19.85715294** | **ERCC4** | **0.1** |
| **DLX3** | **19.74795914** | **ERCC5** | **0.1** |
| **OTOA** | **19.7059536** | **TINF2** | **0.1** |
| **LMX1A** | **19.68141937** | **ABCD1** | **0.1** |
| **MT-ND4** | **19.57990646** | **ABCA12** | **0.1** |
| **PKP1** | **19.57672501** | **EDAR** | **0.1** |
| **KRT16** | **19.56599808** | **AUTS2** | **0.1** |
| **ATP2B2** | **19.54515648** | **FGF5** | **0.1** |
| **ABCA4** | **19.49136734** | **FGFR1** | **0.1** |
| **SMARCA4** | **19.45989037** | **VSIG4** | **0.1** |
| **AARS1** | **19.44321632** | **HRAS** | **0.1** |
| **SKIC2** | **19.38719749** | **DDB2** | **0.1** |
| **TMCO1** | **19.37694168** | **HLCS** | **0.1** |
| **DCAF17** | **19.32540894** | **ADA** | **0.1** |
| **LEF1** | **19.31134415** | **MTND1P31** | **0.1** |
| **MBTPS2** | **19.29798508** | **PUDPP2** | **0.1** |
| **TPRN** | **19.24631119** | **SPPL2C** | **0.1** |
| **HLA-DRB1** | **19.23718643** | **HOXD-AS2** | **0.1** |
| **DKC1** | **19.23387909** | **TMEM74** | **0.1** |
| **KRT32** | **19.23063469** | **DRAIC** | **0.1** |
| **PEX1** | **19.18429947** | **MKLN1-AS** | **0.1** |
| **SERPINB6** | **19.15555382** | **ZBED6** | **0.1** |
| **WRN** | **19.13678741** | **ALOX12B** | **0.1** |
| **PROM1** | **19.07913208** | **ZFHX4-AS1** | **0.1** |
| **ERBB2** | **19.05173302** | **LINC01432** | **0.1** |
| **P2RX2** | **19.04777718** | **RNU4ATAC** | **0.1** |
| **DMXL2** | **18.99095535** | **MAPT-AS1** | **0.1** |
| **LRP2** | **18.97895622** | **APCDD1** | **0.1** |
| **VDR** | **18.83842087** | **DKC1** | **0.1** |
| **PSEN1** | **18.79802513** | **HLA-DRA** | **0.1** |
| **PNPLA6** | **18.76371956** | **WARS2-AS1** | **0.1** |
| **SKIC3** | **18.67868614** | **RORA-AS1** | **0.1** |
| **COL2A1** | **18.63652611** | **EDARADD** | **0.1** |
| **OFD1** | **18.61568451** | **LINC02745** | **0.1** |
| **AXIN2** | **18.60719299** | **KLF15** | **0.1** |
| **CREBBP** | **18.59455681** | **LINC01494** | **0.1** |
| **GNRH1** | **18.55688858** | **COL3A1** | **0.1** |
| **KREMEN1** | **18.53718948** | **COL7A1** | **0.1** |
| **KRT1** | **18.4973793** | **PNPLA1** | **0.1** |
| **ARID1A** | **18.48803139** | **EOGT** | **0.1** |
| **FLNA** | **18.46179199** | **AADACL2-AS1** | **0.1** |
| **POLR1C** | **18.43649483** | **HFE** | **0.1** |
| **PTPN22** | **18.4170475** |  |  |
| **FAS** | **18.41568756** |  |  |
| **BRCA2** | **18.34498024** |  |  |
| **SMARCB1** | **18.29851341** |  |  |
| **LEP** | **18.23626709** |  |  |
| **DSG1** | **18.23062897** |  |  |
| **NECTIN1** | **18.2063694** |  |  |
| **MEN1** | **18.19177055** |  |  |
| **SHH** | **18.17341232** |  |  |
| **BMP4** | **18.09673309** |  |  |
| **CLCNKA** | **18.0439949** |  |  |
| **NMNAT1** | **18.02834892** |  |  |
| **KRT33B** | **17.97627449** |  |  |
| **GNAS** | **17.94613647** |  |  |
| **CDKL5** | **17.87902451** |  |  |
| **TARS1** | **17.87370872** |  |  |
| **BTD** | **17.83979988** |  |  |
| **SMC1A** | **17.81448364** |  |  |
| **SLC24A4** | **17.81031418** |  |  |
| **BDNF** | **17.71781921** |  |  |
| **KRT5** | **17.70976448** |  |  |
| **SMO** | **17.62594795** |  |  |
| **OTOGL** | **17.45627022** |  |  |
| **SLC33A1** | **17.41577339** |  |  |
| **PTCH1** | **17.40189934** |  |  |
| **TINF2** | **17.39777374** |  |  |
| **MLPH** | **17.36451149** |  |  |
| **SOX2** | **17.3412075** |  |  |
| **ALB** | **17.33842659** |  |  |
| **NOTCH2** | **17.27212906** |  |  |
| **GGPS1** | **17.2501812** |  |  |
| **RPGR** | **17.16576385** |  |  |
| **TWIST2** | **17.14744949** |  |  |
| **CCND1** | **17.12138939** |  |  |
| **IL1B** | **17.080616** |  |  |
| **ERCC8** | **17.07812119** |  |  |
| **SREBF1** | **17.05097008** |  |  |
| **MAP2K2** | **17.05093193** |  |  |
| **TMEM132E** | **17.02045441** |  |  |
| **DIAPH3** | **17.0202713** |  |  |
| **SLC24A5** | **17.0088501** |  |  |
| **TERC** | **16.93831062** |  |  |
| **RNF113A** | **16.89575577** |  |  |
| **LAMA3** | **16.87504005** |  |  |
| **KRT33A** | **16.87442398** |  |  |
| **AFG3L2** | **16.83367538** |  |  |
| **NOTCH3** | **16.81316757** |  |  |
| **COL4A5** | **16.81156921** |  |  |
| **NTRK1** | **16.80509949** |  |  |
| **DNMT3A** | **16.77902222** |  |  |
| **IGF2** | **16.77228165** |  |  |
| **HLA-DQB1** | **16.75554085** |  |  |
| **KRT10** | **16.68902206** |  |  |
| **HGSNAT** | **16.66703987** |  |  |
| **PROKR2** | **16.64989853** |  |  |
| **ACTB** | **16.64893341** |  |  |
| **MAPT** | **16.64187431** |  |  |
| **GTF2E2** | **16.6311245** |  |  |
| **SLITRK1** | **16.6287899** |  |  |
| **APC** | **16.61570168** |  |  |
| **CEP290** | **16.56897736** |  |  |
| **LAMC2** | **16.50523758** |  |  |
| **EXT1** | **16.47920609** |  |  |
| **SLC44A4** | **16.43877411** |  |  |
| **TCOF1** | **16.41901207** |  |  |
| **SDHA** | **16.39703178** |  |  |
| **ERCC4** | **16.37891579** |  |  |
| **GPSM2** | **16.36401749** |  |  |
| **KCNE1** | **16.35736084** |  |  |
| **MTOR** | **16.33996773** |  |  |
| **ANXA5** | **16.33410645** |  |  |
| **TGM1** | **16.32990074** |  |  |
| **ARSG** | **16.32738686** |  |  |
| **RIPK4** | **16.30848312** |  |  |
| **SDHB** | **16.30801964** |  |  |
| **EIF2AK3** | **16.30210114** |  |  |
| **TWNK** | **16.29223633** |  |  |
| **POLR2F** | **16.28576088** |  |  |
| **ESR1** | **16.28302002** |  |  |
| **HLA-B** | **16.2804718** |  |  |
| **HOXC13-AS** | **16.27881432** |  |  |
| **BRF1** | **16.27782059** |  |  |
| **GJB4** | **16.27678871** |  |  |
| **PRPH2** | **16.26972198** |  |  |
| **NARS2** | **16.24047089** |  |  |
| **MAFB** | **16.22927284** |  |  |
| **MPZ** | **16.1708622** |  |  |
| **NSD1** | **16.1567688** |  |  |
| **SPTLC1** | **16.15468597** |  |  |
| **PKHD1** | **16.15192032** |  |  |
| **LORICRIN** | **16.14395142** |  |  |
| **PDGFRB** | **16.13586426** |  |  |
| **CAV1** | **16.1218586** |  |  |
| **HTT** | **16.09500122** |  |  |
| **EPS8L2** | **16.05617905** |  |  |
| **KRT6A** | **16.04844475** |  |  |
| **ILDR1** | **16.03631783** |  |  |
| **COL9A1** | **15.98451424** |  |  |
| **PLS1** | **15.97119141** |  |  |
| **OSBPL2** | **15.97008705** |  |  |
| **GJB1** | **15.96668816** |  |  |
| **NLRP3** | **15.94778442** |  |  |
| **MTHFR** | **15.77179623** |  |  |
| **FGF8** | **15.76226425** |  |  |
| **MIR96** | **15.68143082** |  |  |
| **DUSP6** | **15.67845154** |  |  |
| **LRP5** | **15.67008591** |  |  |
| **DCT** | **15.62502384** |  |  |
| **WDR11** | **15.61032772** |  |  |
| **PERP** | **15.59153366** |  |  |
| **MT-TK** | **15.57411385** |  |  |
| **CTLA4** | **15.57320595** |  |  |
| **IFNG** | **15.56565762** |  |  |
| **CBS** | **15.56213284** |  |  |
| **INSR** | **15.55187416** |  |  |
| **RECQL4** | **15.55032063** |  |  |
| **TNFSF11** | **15.52797318** |  |  |
| **FMR1** | **15.51664448** |  |  |
| **RBM28** | **15.45734787** |  |  |
| **KRT82** | **15.4568634** |  |  |
| **KRT15** | **15.43126392** |  |  |
| **BAP1** | **15.42777061** |  |  |
| **VPS13B** | **15.3968277** |  |  |
| **VHL** | **15.39025307** |  |  |
| **UBE3A** | **15.37771416** |  |  |
| **ALX4** | **15.34662819** |  |  |
| **PLEC** | **15.32793045** |  |  |
| **ELN** | **15.32323265** |  |  |
| **SOD1** | **15.31793022** |  |  |
| **PNPT1** | **15.28510761** |  |  |
| **NOG** | **15.28407669** |  |  |
| **BBS1** | **15.21458054** |  |  |
| **RAF1** | **15.20219421** |  |  |
| **VEGFA** | **15.19202423** |  |  |
| **TRMU** | **15.17615604** |  |  |
| **ATP6V1B2** | **15.16219044** |  |  |
| **MDM2** | **15.1102066** |  |  |
| **SMARCE1** | **15.10320568** |  |  |
| **WDR19** | **15.09733105** |  |  |
| **ELMOD3** | **15.08744621** |  |  |
| **GLI2** | **15.08349228** |  |  |
| **GAN** | **15.03503132** |  |  |
| **TMC2** | **15.0344677** |  |  |
| **PRKAR1A** | **14.99577904** |  |  |
| **GNRHR** | **14.94462681** |  |  |
| **PBX1** | **14.90750885** |  |  |
| **MCM2** | **14.88234806** |  |  |
| **FGF17** | **14.87486458** |  |  |
| **WT1** | **14.86753654** |  |  |
| **MAPK1** | **14.86406517** |  |  |
| **CCDC47** | **14.85377979** |  |  |
| **SPRY4** | **14.8196888** |  |  |
| **FIG4** | **14.81314659** |  |  |
| **SETD2** | **14.8020401** |  |  |
| **MT-ATP6** | **14.79719162** |  |  |
| **CCDC50** | **14.78764248** |  |  |
| **FGF5** | **14.775177** |  |  |
| **TPCN2** | **14.77444363** |  |  |
| **NECTIN4** | **14.77070427** |  |  |
| **ANOS1** | **14.72203827** |  |  |
| **IFT172** | **14.69748497** |  |  |
| **CP** | **14.69129944** |  |  |
| **ROR1** | **14.68733788** |  |  |
| **VCP** | **14.68169403** |  |  |
| **NAGLU** | **14.65981865** |  |  |
| **BMP2** | **14.64147949** |  |  |
| **ABCD1** | **14.62238407** |  |  |
| **IGF1R** | **14.5948143** |  |  |
| **KRT84** | **14.56614971** |  |  |
| **NPM1** | **14.55853653** |  |  |
| **TSC2** | **14.54997921** |  |  |
| **MLH1** | **14.54660606** |  |  |
| **ASXL1** | **14.48850441** |  |  |
| **POLR3A** | **14.4740696** |  |  |
| **SYNE4** | **14.4732132** |  |  |
| **RHO** | **14.47197437** |  |  |
| **RAI1** | **14.44603539** |  |  |
| **NBN** | **14.44483185** |  |  |
| **SOS1** | **14.42310715** |  |  |
| **MIA3** | **14.36822796** |  |  |
| **SOST** | **14.32987309** |  |  |
| **NF2** | **14.31751442** |  |  |
| **LAMB3** | **14.31005287** |  |  |
| **STAT3** | **14.30378342** |  |  |
| **ATRX** | **14.28984642** |  |  |
| **GUCA1A** | **14.2820034** |  |  |
| **SLC25A24** | **14.27250862** |  |  |
| **FLCN** | **14.24683762** |  |  |
| **ALOXE3** | **14.24185848** |  |  |
| **NIPBL** | **14.23839092** |  |  |
| **MARS1** | **14.23390198** |  |  |
| **RPE65** | **14.22383595** |  |  |
| **FRAS1** | **14.20849514** |  |  |
| **BSND** | **14.20364761** |  |  |
| **ABCC9** | **14.16443634** |  |  |
| **PROK2** | **14.1547184** |  |  |
| **WNT10B** | **14.12896633** |  |  |
| **HFE** | **14.09335423** |  |  |
| **TFAP2A** | **14.09234238** |  |  |
| **PRKN** | **14.07451916** |  |  |
| **HRURF** | **14.03351212** |  |  |
| **TNFRSF11A** | **14.01464939** |  |  |
| **CLIC5** | **14.01404667** |  |  |
| **TWIST1** | **14.00600243** |  |  |
| **GATA3** | **13.99818802** |  |  |
| **BSCL2** | **13.98591042** |  |  |
| **PPP2R3C** | **13.96778488** |  |  |
| **CTSC** | **13.96333885** |  |  |
| **MT-ND6** | **13.95372772** |  |  |
| **EPS8** | **13.89075279** |  |  |
| **SCN5A** | **13.88175583** |  |  |
| **COL7A1** | **13.83881569** |  |  |
| **EPRS1** | **13.8350563** |  |  |
| **NFKBIA** | **13.82161808** |  |  |
| **COL1A1** | **13.8173008** |  |  |
| **ALOX12B** | **13.81123543** |  |  |
| **SNCA** | **13.78027916** |  |  |
| **HPS3** | **13.76745033** |  |  |
| **GUCY2D** | **13.75975037** |  |  |
| **PARN** | **13.75943375** |  |  |
| **CRYM** | **13.75467968** |  |  |
| **CTCF** | **13.7509613** |  |  |
| **MT-TH** | **13.71840477** |  |  |
| **SRD5A2** | **13.70414829** |  |  |
| **BCL2** | **13.69364071** |  |  |
| **MT-ND5** | **13.68224144** |  |  |
| **CLDN1** | **13.65757942** |  |  |
| **COL1A2** | **13.63669109** |  |  |
| **FBN1** | **13.61964607** |  |  |
| **TSHR** | **13.61632538** |  |  |
| **TSC1** | **13.61234283** |  |  |
| **GRN** | **13.59071445** |  |  |
| **USP53** | **13.5777359** |  |  |
| **ABCC1** | **13.55056095** |  |  |
| **TACR3** | **13.51930428** |  |  |
| **LZTR1** | **13.50937653** |  |  |
| **SCO2** | **13.50500679** |  |  |
| **SLITRK6** | **13.49905777** |  |  |
| **DIABLO** | **13.4975481** |  |  |
| **MIR34A** | **13.466012** |  |  |
| **GATA1** | **13.44112015** |  |  |
| **CLCN7** | **13.42457962** |  |  |
| **ST14** | **13.42412758** |  |  |
| **PRL** | **13.42152119** |  |  |
| **CHM** | **13.40449142** |  |  |
| **EGR2** | **13.39814472** |  |  |
| **HPS5** | **13.37795353** |  |  |
| **EPS8L3** | **13.37761307** |  |  |
| **HOMER2** | **13.37612247** |  |  |
| **LIG4** | **13.37218285** |  |  |
| **CLN3** | **13.36243057** |  |  |
| **PRNP** | **13.35899448** |  |  |
| **APP** | **13.33577156** |  |  |
| **TRPV3** | **13.33257484** |  |  |
| **KRT6B** | **13.32169437** |  |  |
| **RRM2B** | **13.31456375** |  |  |
| **AP1B1** | **13.30367756** |  |  |
| **IL1A** | **13.29749489** |  |  |
| **MAF** | **13.29112816** |  |  |
| **RIN2** | **13.26453876** |  |  |
| **MPZL2** | **13.25038624** |  |  |
| **TMEM43** | **13.24702263** |  |  |
| **ADIPOQ** | **13.24332237** |  |  |
| **KRT72** | **13.24268818** |  |  |
| **GBA1** | **13.22980595** |  |  |
| **HARS2** | **13.22797394** |  |  |
| **RAB33A** | **13.22586346** |  |  |
| **SLC52A2** | **13.22373104** |  |  |
| **FGF10** | **13.22097969** |  |  |
| **FOXC1** | **13.2209301** |  |  |
| **COL3A1** | **13.21118355** |  |  |
| **RTEL1** | **13.17376995** |  |  |
| **PMP22** | **13.17327499** |  |  |
| **MAP1B** | **13.16923809** |  |  |
| **KMT2A** | **13.168787** |  |  |
| **CTC1** | **13.14969635** |  |  |
| **CRX** | **13.13882732** |  |  |
| **EP300** | **13.08419991** |  |  |
| **TRPV4** | **13.06714249** |  |  |
| **FKBP14** | **13.06566334** |  |  |
| **HDAC8** | **13.05507183** |  |  |
| **ATOH1** | **13.04493237** |  |  |
| **MT-CYB** | **13.04342842** |  |  |
| **PSORS1C1** | **13.02780628** |  |  |
| **IFIH1** | **13.01610184** |  |  |
| **HSD17B4** | **13.0118885** |  |  |
| **AIRE** | **13.01123047** |  |  |
| **MED12** | **12.99598503** |  |  |
| **MT-TS2** | **12.99213791** |  |  |
| **ABCC8** | **12.98824596** |  |  |
| **IRF6** | **12.97827625** |  |  |
| **TGFBR2** | **12.97171688** |  |  |
| **SURF1** | **12.92770958** |  |  |
| **NR5A1** | **12.92438602** |  |  |
| **RLBP1** | **12.89561272** |  |  |
| **CRP** | **12.8939991** |  |  |
| **RNU4ATAC** | **12.88220024** |  |  |
| **CSTB** | **12.88071442** |  |  |
| **TARDBP** | **12.87397575** |  |  |
| **KCTD1** | **12.86373138** |  |  |
| **KRT19** | **12.8635416** |  |  |
| **TCF4** | **12.8558445** |  |  |
| **REST** | **12.85270786** |  |  |
| **DSG3** | **12.83636951** |  |  |
| **NEFL** | **12.80160904** |  |  |
| **FOS** | **12.79911041** |  |  |
| **GNPTAB** | **12.78294373** |  |  |
| **NR2E3** | **12.78017139** |  |  |
| **TBL1X** | **12.74237823** |  |  |
| **CABP2** | **12.74183655** |  |  |
| **WWOX** | **12.74121094** |  |  |
| **NOS3** | **12.72925663** |  |  |
| **KCNMA1** | **12.71256733** |  |  |
| **DSG2** | **12.70432568** |  |  |
| **FEZF1** | **12.67978096** |  |  |
| **FGF7** | **12.66241646** |  |  |
| **HPS6** | **12.65537834** |  |  |
| **C9orf72** | **12.64627838** |  |  |
| **BNC1** | **12.63440514** |  |  |
| **LOC108167315** | **12.62312031** |  |  |
| **NTNG1** | **12.61871624** |  |  |
| **ITGA6** | **12.61695671** |  |  |
| **GUSB** | **12.58997917** |  |  |
| **ARID2** | **12.58946419** |  |  |
| **SHBG** | **12.57963085** |  |  |
| **TPP1** | **12.57676315** |  |  |
| **PISD** | **12.56585693** |  |  |
| **KRT73** | **12.56207561** |  |  |
| **LYST** | **12.56057739** |  |  |
| **MYC** | **12.54708862** |  |  |
| **CYP19A1** | **12.52780342** |  |  |
| **STK11** | **12.52645493** |  |  |
| **MSH2** | **12.51728153** |  |  |
| **TULP1** | **12.50833893** |  |  |
| **MIR21** | **12.49573326** |  |  |
| **ITGB6** | **12.48604393** |  |  |
| **TTR** | **12.48224545** |  |  |
| **BEST1** | **12.47277355** |  |  |
| **MSX2** | **12.46565437** |  |  |
| **ODC1** | **12.46463108** |  |  |
| **BANF1** | **12.45856476** |  |  |
| **HLA-DQA1** | **12.44658089** |  |  |
| **NR3C1** | **12.44483757** |  |  |
| **MTX2** | **12.43388844** |  |  |
| **NDUFAF2** | **12.43287659** |  |  |
| **SNRPN** | **12.42512226** |  |  |
| **NOP10** | **12.42291069** |  |  |
| **ADCY1** | **12.39028358** |  |  |
| **HLA-A** | **12.34999275** |  |  |
| **CDH23-AS1** | **12.33993912** |  |  |
| **CDK4** | **12.30393028** |  |  |
| **TIMM8A** | **12.29674435** |  |  |
| **CNGB3** | **12.26308632** |  |  |
| **SCN1A** | **12.26220703** |  |  |
| **CDKN1A** | **12.23201466** |  |  |
| **MUTYH** | **12.21707726** |  |  |
| **PIK3R1** | **12.20983219** |  |  |
| **NFKB1** | **12.19803047** |  |  |
| **VAC14** | **12.19669342** |  |  |
| **AHSG** | **12.1842308** |  |  |
| **RPS19** | **12.13869667** |  |  |
| **GLI1** | **12.11667347** |  |  |
| **KISS1** | **12.11279297** |  |  |
| **HLA-DRA** | **12.10452652** |  |  |
| **MMP2** | **12.09466839** |  |  |
| **GATA4** | **12.09144592** |  |  |
| **H2AC18** | **12.08889771** |  |  |
| **BBS2** | **12.0855484** |  |  |
| **SMC3** | **12.08512878** |  |  |
| **CACNA1A** | **12.07550907** |  |  |
| **CBL** | **12.07239532** |  |  |
| **KRT34** | **12.06860447** |  |  |
| **SGSH** | **12.06724739** |  |  |
| **SQSTM1** | **12.06245422** |  |  |
| **EZH2** | **12.0595789** |  |  |
| **CRB1** | **12.05801296** |  |  |
| **SOX3** | **12.05339432** |  |  |
| **ABCA12** | **12.00193596** |  |  |
| **DPH2** | **11.97052193** |  |  |
| **MERTK** | **11.96746349** |  |  |
| **CDKN1B** | **11.96605682** |  |  |
| **PSMB8** | **11.94139862** |  |  |
| **FUS** | **11.93724823** |  |  |
| **ITGB1** | **11.92770386** |  |  |
| **UBR1** | **11.92213058** |  |  |
| **CACNA1F** | **11.90140533** |  |  |
| **DICER1** | **11.87345028** |  |  |
| **CYP17A1** | **11.87091923** |  |  |
| **EGF** | **11.82675362** |  |  |
| **TUBB1** | **11.8056612** |  |  |
| **MAP3K7** | **11.80196571** |  |  |
| **CASR** | **11.80174065** |  |  |
| **S1PR2** | **11.79596806** |  |  |
| **GHRL** | **11.78632927** |  |  |
| **KISS1R** | **11.78311348** |  |  |
| **SPP1** | **11.77736664** |  |  |
| **PRPF8** | **11.7647934** |  |  |
| **MKS1** | **11.76184177** |  |  |
| **HPS1** | **11.75636196** |  |  |
| **DNMT3B** | **11.73900223** |  |  |
| **FREM2** | **11.73731422** |  |  |
| **TGFB2** | **11.73134518** |  |  |
| **NHP2** | **11.73111534** |  |  |
| **NSUN2** | **11.72866249** |  |  |
| **RAD21** | **11.72797775** |  |  |
| **ZBTB20** | **11.72738266** |  |  |
| **ADAM17** | **11.71309853** |  |  |
| **CD36** | **11.70398521** |  |  |
| **RAC1** | **11.68834591** |  |  |
| **KLHL7** | **11.67940521** |  |  |
| **PPT1** | **11.6757555** |  |  |
| **ERCC1** | **11.66971397** |  |  |
| **CASP8** | **11.66913605** |  |  |
| **AHI1** | **11.64101982** |  |  |
| **FOXP4** | **11.63463306** |  |  |
| **MMP1** | **11.62976646** |  |  |
| **SPTLC2** | **11.61929226** |  |  |
| **AA1** | **11.59422874** |  |  |
| **BBS10** | **11.57687569** |  |  |
| **IFT43** | **11.57470703** |  |  |
| **KAT6B** | **11.57441807** |  |  |
| **FAT4** | **11.52331734** |  |  |
| **TRRAP** | **11.52103043** |  |  |
| **BLOC1S5** | **11.51599789** |  |  |
| **IL2** | **11.50484467** |  |  |
| **PRPF31** | **11.50313187** |  |  |
| **CFAP418** | **11.5016098** |  |  |
| **EDNRA** | **11.48332596** |  |  |
| **B2M** | **11.48097897** |  |  |
| **CYLD** | **11.48092461** |  |  |
| **BMPR1A** | **11.47973633** |  |  |
| **SETBP1** | **11.47806454** |  |  |
| **PDGFRA** | **11.47167587** |  |  |
| **FOXG1** | **11.46868134** |  |  |
| **ATP2A2** | **11.46408844** |  |  |
| **MYO1C** | **11.45827675** |  |  |
| **CRYAA** | **11.45777512** |  |  |
| **PAH** | **11.45655346** |  |  |
| **CTSD** | **11.45251656** |  |  |
| **FOXP1** | **11.43920326** |  |  |
| **DSCAS** | **11.42404461** |  |  |
| **TNFRSF11B** | **11.40724468** |  |  |
| **EPG5** | **11.40563774** |  |  |
| **FHIT** | **11.40388393** |  |  |
| **RASA2** | **11.39717388** |  |  |
| **FA2H** | **11.3968544** |  |  |
| **LRRK2** | **11.39607906** |  |  |
| **HTRA1** | **11.38297272** |  |  |
| **TREM2** | **11.3798542** |  |  |
| **IFT122** | **11.37759399** |  |  |
| **DNAJC6** | **11.37346268** |  |  |
| **DACT1** | **11.37011528** |  |  |
| **S100A3** | **11.36822605** |  |  |
| **CLCNKB** | **11.33970737** |  |  |
| **ASL** | **11.32448959** |  |  |
| **ALDH18A1** | **11.32105827** |  |  |
| **TGFB3** | **11.31929207** |  |  |
| **GRXCR2** | **11.29957676** |  |  |
| **IL1RN** | **11.29289341** |  |  |
| **PRPF3** | **11.28305244** |  |  |
| **MIR29A** | **11.28117085** |  |  |
| **GREB1L** | **11.26263618** |  |  |
| **CD164** | **11.25531673** |  |  |
| **CLRN1-AS1** | **11.25336742** |  |  |
| **MAGEL2** | **11.25265503** |  |  |
| **WRAP53** | **11.24553871** |  |  |
| **CTNND1** | **11.2372694** |  |  |
| **RAD51** | **11.22953796** |  |  |
| **TLR4** | **11.22062969** |  |  |
| **ENPP1** | **11.21395874** |  |  |
| **TJP2** | **11.21352482** |  |  |
| **CDKN1C** | **11.21191978** |  |  |
| **FSCN2** | **11.2058363** |  |  |
| **KRT37** | **11.20367146** |  |  |
| **IKZF1** | **11.20121765** |  |  |
| **GALC** | **11.19868469** |  |  |
| **TIMP3** | **11.19593334** |  |  |
| **TYMS** | **11.18949699** |  |  |
| **MT-CO3** | **11.1837616** |  |  |
| **PLA2G6** | **11.15466499** |  |  |
| **BLM** | **11.14980507** |  |  |
| **GSTM1** | **11.14705086** |  |  |
| **CD4** | **11.13868046** |  |  |
| **IRX5** | **11.12704945** |  |  |
| **SOX4** | **11.12703609** |  |  |
| **NR0B1** | **11.11391926** |  |  |
| **ASIC5** | **11.09444714** |  |  |
| **MFSD8** | **11.09115887** |  |  |
| **ARSA** | **11.0613718** |  |  |
| **BRIP1** | **11.06097412** |  |  |
| **PANK2** | **11.05308533** |  |  |
| **CERS3** | **11.04617119** |  |  |
| **GDF6** | **11.01662922** |  |  |
| **KIF11** | **11.00602055** |  |  |
| **SEMA4A** | **10.99856758** |  |  |
| **PKP2** | **10.99749279** |  |  |
| **PAX6** | **10.99284172** |  |  |
| **PAX2** | **10.98482418** |  |  |
| **FLVCR1** | **10.98019218** |  |  |
| **CLRN2** | **10.9737606** |  |  |
| **SLC52A3** | **10.97339249** |  |  |
| **HARS1** | **10.9612608** |  |  |
| **RPGRIP1** | **10.95240307** |  |  |
| **KIF1A** | **10.95114613** |  |  |
| **MSRB3** | **10.9508543** |  |  |
| **ZNF469** | **10.94051743** |  |  |
| **SMCHD1** | **10.9402256** |  |  |
| **FLRT3** | **10.9274416** |  |  |
| **FGF3** | **10.92657948** |  |  |
| **RUNX1** | **10.91906548** |  |  |
| **BMP6** | **10.9160471** |  |  |
| **ATXN2** | **10.91593742** |  |  |
| **GRAP** | **10.90226364** |  |  |
| **CASP3** | **10.90160561** |  |  |
| **DTNBP1** | **10.89889145** |  |  |
| **TPO** | **10.8970356** |  |  |
| **WBP2** | **10.87553501** |  |  |
| **MIR17** | **10.86757183** |  |  |
| **STAT4** | **10.86715221** |  |  |
| **CDKN2B** | **10.85928535** |  |  |
| **ATRIP** | **10.82064819** |  |  |
| **FLNC** | **10.80220795** |  |  |
| **AP1S1** | **10.80180359** |  |  |
| **SOS2** | **10.80051327** |  |  |
| **EFNB1** | **10.79495049** |  |  |
| **ALPL** | **10.78869534** |  |  |
| **HDAC4** | **10.77315998** |  |  |
| **WNT5A** | **10.76519775** |  |  |
| **RIMS1** | **10.76225948** |  |  |
| **LIPC** | **10.75429726** |  |  |
| **OCRL** | **10.75210285** |  |  |
| **NEU1** | **10.74261761** |  |  |
| **TYMP** | **10.740098** |  |  |
| **CD34** | **10.73546028** |  |  |
| **CHN1** | **10.73463058** |  |  |
| **KRTAP8-1** | **10.72554111** |  |  |
| **AP3D1** | **10.72263145** |  |  |
| **RYR2** | **10.71842384** |  |  |
| **ATL1** | **10.68859673** |  |  |
| **FASLG** | **10.68715763** |  |  |
| **IL2RA** | **10.68457222** |  |  |
| **AGK** | **10.67647076** |  |  |
| **BBS4** | **10.67309952** |  |  |
| **RBBP8** | **10.66401291** |  |  |
| **ABL1** | **10.65764999** |  |  |
| **ENG** | **10.65736771** |  |  |
| **YAP1** | **10.6248188** |  |  |
| **CFH** | **10.62327862** |  |  |
| **EDNRB-AS1** | **10.61365318** |  |  |
| **SNRNP200** | **10.61156082** |  |  |
| **TOPORS** | **10.61120033** |  |  |
| **PEX5** | **10.60965824** |  |  |
| **POLR1D** | **10.60961342** |  |  |
| **ESR2** | **10.60307693** |  |  |
| **CLPP** | **10.59008884** |  |  |
| **C19orf12** | **10.57486057** |  |  |
| **HOXB1** | **10.57103062** |  |  |
| **IL17A** | **10.56010628** |  |  |
| **CDH11** | **10.55919266** |  |  |
| **USB1** | **10.55056572** |  |  |
| **ANKRD11** | **10.54498768** |  |  |
| **PQBP1** | **10.54008293** |  |  |
| **LMX1B** | **10.51515961** |  |  |
| **LOC109504725** | **10.51116085** |  |  |
| **CCBE1** | **10.50289154** |  |  |
| **CDHR1** | **10.50004387** |  |  |
| **SPRED1** | **10.49753761** |  |  |
| **SMPD1** | **10.48987007** |  |  |
| **SRY** | **10.48555088** |  |  |
| **C11orf65** | **10.4839983** |  |  |
| **ZFYVE26** | **10.475214** |  |  |
| **STN1** | **10.46442699** |  |  |
| **KCNJ11** | **10.45865154** |  |  |
| **BDP1** | **10.43777847** |  |  |
| **BBIP1** | **10.43446732** |  |  |
| **DNAH8** | **10.43391132** |  |  |
| **SDHC** | **10.43236256** |  |  |
| **PDE6B** | **10.42798138** |  |  |
| **ITGA3** | **10.42558384** |  |  |
| **BAX** | **10.42115498** |  |  |
| **PPIP5K2** | **10.4175005** |  |  |
| **DPF2** | **10.41728687** |  |  |
| **IDS** | **10.41162491** |  |  |
| **NPC1** | **10.4075737** |  |  |
| **AA2** | **10.39135933** |  |  |
| **TXNL4A** | **10.39123917** |  |  |
| **SPNS2** | **10.36913204** |  |  |
| **LMNB2** | **10.35918236** |  |  |
| **CARD14** | **10.35316753** |  |  |
| **TCF7L2** | **10.34921455** |  |  |
| **TRNT1** | **10.33467865** |  |  |
| **KRT27** | **10.3287878** |  |  |
| **ANAPC15** | **10.32698154** |  |  |
| **CCDC141** | **10.31692028** |  |  |
| **FOXQ1** | **10.31320667** |  |  |
| **KRTAP13-1** | **10.30816269** |  |  |
| **FTL** | **10.2985096** |  |  |
| **MSH6** | **10.29452801** |  |  |
| **NPHP1** | **10.2784481** |  |  |
| **CHEK2** | **10.27124023** |  |  |
| **ZPR1** | **10.26770306** |  |  |
| **YY1** | **10.26657867** |  |  |
| **ADA2** | **10.26374245** |  |  |
| **OTX2** | **10.26221657** |  |  |
| **CLN6** | **10.2608633** |  |  |
| **WDR72** | **10.25871468** |  |  |
| **SYP** | **10.25358772** |  |  |
| **PORCN** | **10.23394489** |  |  |
| **PEX13** | **10.23356724** |  |  |
| **SUFU** | **10.22554207** |  |  |
| **DKK1** | **10.22358322** |  |  |
| **HPS4** | **10.22298813** |  |  |
| **VCAN** | **10.221488** |  |  |
| **CNGA3** | **10.21897316** |  |  |
| **MKRN3** | **10.21675301** |  |  |
| **AMMECR1** | **10.21399689** |  |  |
| **NRL** | **10.207901** |  |  |
| **RDH12** | **10.20566654** |  |  |
| **IVL** | **10.20529652** |  |  |
| **PDE6A** | **10.20355988** |  |  |
| **ARL3** | **10.19335747** |  |  |
| **TREX1** | **10.19266796** |  |  |
| **SMARCC2** | **10.17970657** |  |  |
| **HS6ST1** | **10.1741066** |  |  |
| **JAK2** | **10.16441345** |  |  |
| **GDAP1** | **10.15956974** |  |  |
| **WDR35** | **10.15355778** |  |  |
| **IGF2R** | **10.15016365** |  |  |
| **DNAJC30** | **10.14029884** |  |  |
| **ERCC5** | **10.13594437** |  |  |
| **GATAD1** | **10.1330204** |  |  |
| **PHYH** | **10.12969971** |  |  |
| **TNFRSF1B** | **10.12801933** |  |  |
| **TBK1** | **10.11341286** |  |  |
| **DSC1** | **10.11232376** |  |  |
| **SPG7** | **10.10928059** |  |  |
| **ACOX1** | **10.10578537** |  |  |
| **MYEF2** | **10.08338451** |  |  |
| **FKRP** | **10.06933403** |  |  |
| **PNPLA1** | **10.05458832** |  |  |
| **AIPL1** | **10.04993629** |  |  |
| **IMPG2** | **10.04369354** |  |  |
| **CAT** | **10.04230309** |  |  |
| **NDUFS4** | **10.03635311** |  |  |
| **ZDHHC24** | **10.03029633** |  |  |
| **GATA2** | **10.03010559** |  |  |
| **SF3B4** | **10.01558781** |  |  |
| **EBP** | **10.01290703** |  |  |
| **PALB2** | **10.01136494** |  |  |
